# Glycogen synthase kinase 3 controls migration of the neural crest lineage in mouse and *Xenopus*

**DOI:** 10.1038/s41467-018-03512-5

**Published:** 2018-03-19

**Authors:** Sandra G. Gonzalez Malagon, Anna M. Lopez Muñoz, Daniel Doro, Triòna G. Bolger, Evon Poon, Elizabeth R. Tucker, Hadeel Adel Al-Lami, Matthias Krause, Christopher J. Phiel, Louis Chesler, Karen J. Liu

**Affiliations:** 1 Centre for Craniofacial and Regenerative Biology, King’s College London, London, SE1 9RT UK; 2 Paediatric Solid Tumour Biology, Institute of Cancer Research/Royal Marsden NHS Trust, Surrey, SM2 5NG UK; 3 Randall Division of Cell & Molecular Biophysics, King’s College London, London, SE1 1UL UK; 4 Department of Integrative Biology, University of Colorado Denver, Denver, CO 80204 USA

## Abstract

Neural crest migration is critical to its physiological function. Mechanisms controlling mammalian neural crest migration are comparatively unknown, due to difficulties accessing this cell population in vivo. Here we report requirements of glycogen synthase kinase 3 (GSK3) in regulating the neural crest in *Xenopus* and mouse models. We demonstrate that GSK3 is tyrosine phosphorylated (pY) in mouse neural crest cells and that loss of GSK3 leads to increased pFAK and misregulation of Rac1 and lamellipodin, key regulators of cell migration. Genetic reduction of GSK3 results in failure of migration. We find that pY-GSK3 phosphorylation depends on anaplastic lymphoma kinase (ALK), a protein associated with neuroblastoma. Consistent with this, neuroblastoma cells with increased ALK activity express high levels of pY-GSK3, and blockade of GSK3 or ALK can affect migration of these cells. Altogether, this work identifies a role for GSK3 in cell migration during neural crest development and cancer.

## Abstract (web summary)

Defects in neural crest development cause neurocristopathies and cancer, but what regulates this is unclear. Here, the authors show that glycogen synthase kinase 3 (GSK3) regulates migration of neural crest cells, as shown on genetic deletion of GSK3 in the mouse, and that this acts via anaplastic lymphoma kinase.

## Introduction

The neural crest is a vertebrate-specific, motile population of cells born at the junction of the neural and non-neural ectoderm. This lineage has contributed to our understanding of cellular behaviours, such as contact inhibition of locomotion^1^. It is the origin of many cell types found throughout the organism, including melanocytes, peripheral neurons, cardiac outflow tract and the craniofacial skeleton. Recent reports have highlighted the importance of neural crest cells: their stem-like capacity, their ability to reprogram, to become cancerous, and to drive vertebrate evolution^2,3^. The highly migratory activity of these cells is critical to their in vivo function, not only are their ultimate tissue descendants widespread in the organism but also failure to regulate migration and differentiation in the correct locations is associated with diseases like neuroblastoma (NB)^4–6^. Despite its importance, the specific mechanisms underlying this migratory activity and its control are poorly understood.

In our previous work, we demonstrated a critical role for the pleiotropic kinase glycogen synthase kinase 3 (GSK3) in craniofacial development^7^; therefore, we sought to understand the regulation of GSK3 in neural crest cells, which are integral to most of the craniofacial structures. In vertebrates, the serine/threonine kinase GSK3 is encoded by two paralogous genes, *GSK3α* and *GSK3β*, which are nearly identical throughout their kinase domains^8,9^, and have >100 predicted substrates^9,10^. The effect of GSK3 phosphorylation is substrate dependent and variable, ranging from control of protein degradation (β-catenin, MYC) and localization (nuclear factor of activated T cells) to trafficking (amyloid precursor protein) and cleavage (Gli)^9^.

Given the seemingly ubiquitous expression of GSK3, it is not surprising that fine-tuning of this kinase activity is very complex and not well understood, especially in vivo. GSK3 can be inhibited by N-terminal serine phosphorylations on both GSK3α (serine-21/S21) and GSK3β (serine-9/S9). These serines are targeted by kinases such as protein kinase A and protein kinase B (PKB/AKT) and phosphorylation at these serines prevents GSK3 binding to its substrates^11^. However, interestingly, mouse mutants carrying non-phosphorylatable alanine substitutions at these sites (*GSK3α*^*S21A/S21A*^*; GSK3β*^*S9A/S9A*^) have no obvious developmental phenotypes and are fertile^11^. Using this rigorous approach, functional defects in these animals were limited to regulation of glycogen synthase activity in response to insulin^11^. This evidence, that regulation of GSK3 via PKB-dependent serine phosphorylation is dispensible for embryogenesis, raises the possibility that additional regulatory mechanisms may be important for GSK3 activity in utero. Indeed, inhibition of GSK3 activity by Wnt ligands is serine independent. Instead, inhibition appears to occur via sequestration of GSK3 in response to ligand^12^. This suggests that there exist distinct pools of GSK3 within the cell that are poised for activity.

One positive regulatory mechanism may be via phosphorylation of conserved tyrosine residues (GSK3α-Y279, GSK3β-Y216), which can change the target selectivity of GSK3^13^. While these can be autophosphorylations^14,15^, a recent computational study identified GSK3α as a specific substrate for the kinase ALK, which is often pathologically increased in NB cells^16^. However, these in silico predictions had not been validated in vivo and there had been no studies describing the tissue localization of this phosphorylation. Given the importance of ALK in pathogenesis of neural crest-derived cancers, we considered the possibility that phosphorylation of these tyrosines might be an important mechanism for ALK-dependent tuning of GSK3 activity, which should be specific to the neural crest lineage. Indeed, we identified specific expression of ALK and phospho-tyrosine GSK3 in the delaminating neural crest cells, as well as a requirement for GSK3 in the control of neural crest and NB cell migration.

As noted above, GSK3 is notoriously promiscuous, with many predicted substrates. This has led to reported targets involved in a broad range of biological processes, such as senescence, cell proliferation, axonal outgrowth and signaling. GSK3 is also considered a prime therapeutic target in diverse diseases, such as diabetes, depression, neurodegeneration, cancer and retinitis pigmentosa^17,18^. As a consequence, it is thought that regulation of GSK3 target selection is very context dependent.

Even focussing on the neural crest lineage, GSK3 is thought to have multiple sequential roles beginning with a requirement in patterning of the dorsal axis^19–21^. Based primarily on data from chicken and frog, neural crest-specific GSK3 targets include the Wnt effector β-catenin, the metalloprotease ADAM13 and transcription factors snail and twist^22–24^. Wnt-dependent inhibition of GSK3 is clearly necessary for β-catenin-mediated transcriptional activation during neural crest induction^25^. GSK3 also has proposed roles in the phosphorylation of Twist and Snail, proteins which can regulate the activity and stability of these targets, thus controlling the onset of the epithelial–mesenchymal transition (EMT)^22^. Concurrently, GSK3 interactions with ADAM13 are proposed to be crucial for delamination and entry into the EMT^23^. Given the variety of substrates and the precise timing of development, there is no doubt that GSK3 activity must to be dynamically regulated during neural crest development. However, as noted, mice lacking the inhibitory phosphorylation sites at S21/S9-GSK3α/β have normal craniofacial development and are viable^11^. Therefore, we focus on positive regulation of GSK3 via activating tyrosine phosphorylations.

Here we find a specific activation of GSK3 in neural crest cells as they depart from the neuroepithelium and become migratory mesenchymal cells. This activation is controlled by ALK, which has been implicated in NB and other cancers. Genetic and pharmacological loss of GSK3 activity leads to cytoskeletal changes in migratory neural crest (mNC) cells as well as in NB, raising the possibility that control of GSK3 is a rapid and reversible mechanism for controlling cell migration dynamics in the neural crest lineage.

## Results

### GSK3 is tyrosine phosphorylated in migrating neural crest

**Fig. 1:**
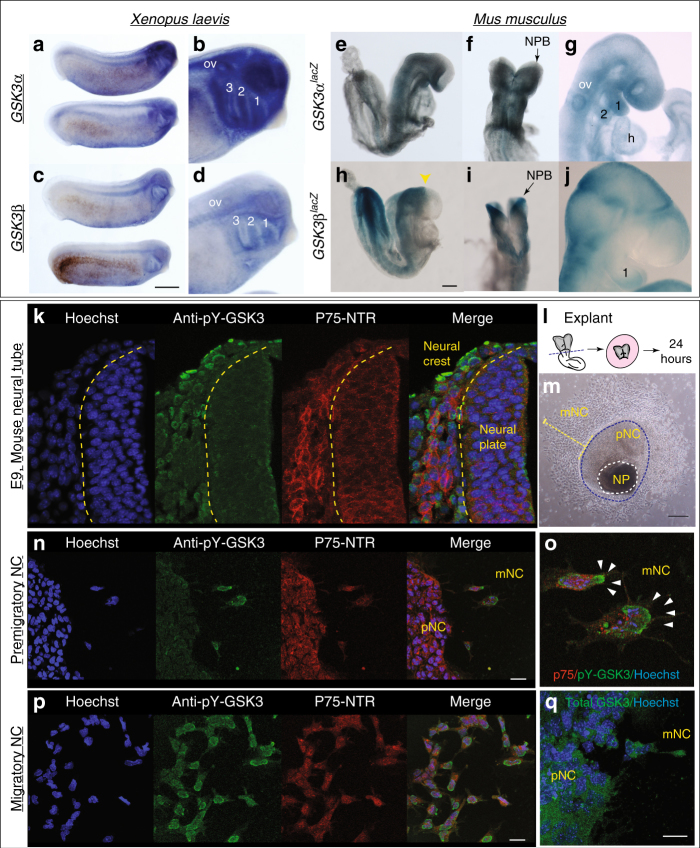
GSK3 genes are expressed during neural crest migration in the frog *Xenopus laevis* and mouse *Mus musculus*. **a**, **b** mRNA in situ hybridization for *Gsk3α* in *X*. *laevis* at stage (st) 25 (**a**) show expression in the pharyngeal pouches, brain, spinal cord and eye vesicle (**b**). **c**, **d** In situ hybridization for *Gsk3β* in *X*. *laevis* at st 25 (**c**). GSK3β is expressed in the pharyngeal pouches and the spinal cord as well as regions of the brain (**d**, scale bar = 0.5 mm). **e**–**g** *GSK3α*^*lacZ*^ is expressed in mice during neural crest migration stages. **e**, **f** In an e8.5 embryo *GSK3α*^*lacZ*^ is expressed in the cephalic mesenchyme, in the neuroepithelium and in the cephalic neural fold. **g** By e9.5–10, *GSK3α*^*lacZ*^ is expressed in the first and second branchial arches (1 and 2) and the frontonasal prominence. **h**–**j** *GSK3β*^*lacZ*^ is expressed in mice when neural crest is actively migrating. **h**, **i** In e8.5 embryos *GSK3β*^*lacZ*^ is mainly expressed in the neuroectoderm, restricted to the prospective hindbrain and some areas in the mesenchyme, scale bar = 200 µm. **j** At e9.5, *GSK3β*^*lacZ*^ is mainly expressed in BA1 and cranial ganglia and in the presumptive trigeminal ganglion. **k**–**p** GSK3α/β are phosphorylated at tyrosines Y216/279 during cranial neural crest cell migration. **k** Transverse cranial section of e9 mouse showing immunoflourescent staining for Hoechst/DNA (blue), pY-GSK3 (green) and p75^NTR^ (neural crest, red). **l** Schematic of e8.5 mouse embryo depicting cranial neural crest (CNC) dissection. **m** Bright-field image of mouse neural crest explant. Two types of cells surround the NP: premigratory neural crest (pNC) cells that are epithelial and migratory neural crest (mNC) scale bar, 250 μm. **n** Cells migrating away from the pNC begin to express pY-GSK3. pNC to the left. All neural crest express p75NTR (red). Note in merge that perinuclear expression of pY-GSK3 is invariably oriented in direction of migration (**o**, white arrowheads). **p** mNC cells express pYGSK3 (green) and p75-NTR (red). **n**,** p** scale bars = 25 µM. **q** Expression of total GSK3 is ubiquitous in pNC and mNC cells. Scale bar = 25 µM. All are representative images from at least three independent experiments

In the embryo, neural crest cells are induced at the neuroepithelial border, subsequently delaminating and becoming migratory. To confirm whether GSK3 was preferentially enriched during neural crest cell migration, we examined mRNA and reporter gene expression for both paralogous genes in frog and mouse and found that these genes were indeed expressed in mNC (Fig. 1a–j). We noted in particular the enrichment of GSK3 expression in the neural plate (NP) stages, at the border of the NP (Fig. 1e, f, h, i) and later on in the mNC cell population, including that destined for the first branchial arch (marked by 1, Fig. 1b, d, g, i). The close protein similarity between GSK3 in frog and mouse, as well as the similar expression patterns, suggests that GSK3 activity may play a conserved role in the vertebrate neural crest.

We were then curious whether GSK3 proteins were activated at specific time points during murine neural crest development. To address this, we used an antibody recognizing a phosphorylated tyrosine in the active site of both GSK3 isoforms (pY279-GSK3α/pY216-GSK3β, referred to hereafter as pY-GSK3). These sites are identical in the two proteins. pY-GSK3 (green) was specifically detected in the cranial neural crest cell population (marked by P75-NTR, red) after emigration from the neural tube (Fig. 1k). This was in contrast to more widespread mRNA expression of GSK3α/β seen above (Fig. 1e–j). This phosphorylation was also confirmed in a simple ex vivo culture system, which allows us to visualize and manipulate specific neural crest populations without complications from surrounding tissues (Fig. 1l). In these assays, NPs from embryonic day 8.5 (e8.5) mouse embryos were explanted and cultured in vitro, prior to neural crest migration, allowing subsequent examination of delaminating neural crest cells. By 24 h of culture, the premigratory neural crest (pNC) cells are spread in an epithelial sheet surrounding the NP, with fully mNC in the outer ring (Fig. 1m). Again, we noted that pY-GSK3 is specifically found in neural crest cells just when they delaminate and become mesenchymal (Fig. 1n–o). In fully mNC cells, the majority of pY-GSK3 appears perinuclear and is invariably oriented in the direction of migration (Fig. 1p). In contrast, staining for total GSK3 appears diffuse and ubiquitous (Fig. 1q).

### Loss of GSK3 prevents migration of cranial neural crest

**Fig. 2:**
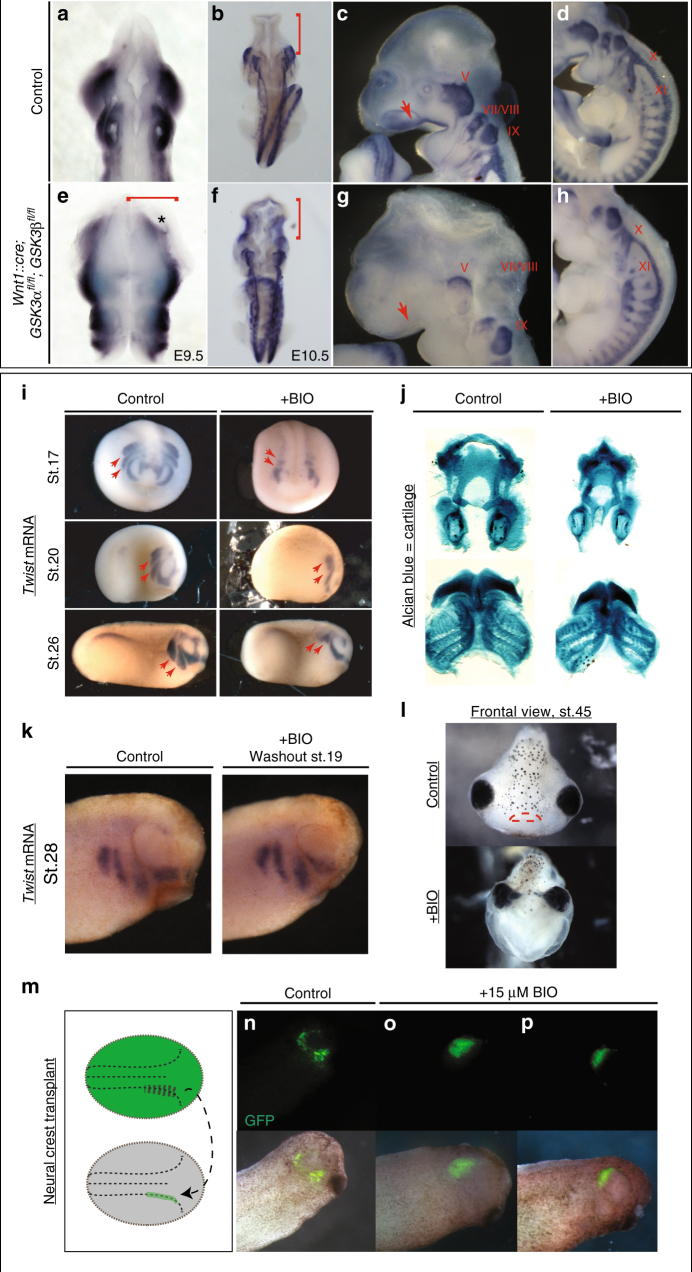
GSK3 is required for neural crest migration in vivo. **a**–**h** mRNA in situ hybridization of *Sox10*, which marks migratory neural crest. **a**, **e** e9.5 mouse embryos (*n* = 3). **b**–**d**, **f**–**h** e10.5 mouse embryos (*n* = 3). **a**, **b**, **e**, **f** Dorsal views. **c**, **d**, **g**, **h** Lateral views of e10.5 control embryos. **a**,** b** In control embryos, *Sox10* expression is absent in the brain (**b**, red bracket) but it is highly expressed along the embryo axis. **e**,** f** Neural crest-specific deletion of GSK3. Dorsal view of e10.5 mutant mouse in which *Sox10* expression has accumulated in the brain (**f**, red bracket). **c**, **d** At e10.5, *Sox10* marks cranial neural crest, which has migrated into the facial prominences (**c**, red arrow) and the cranial ganglia, including the trigeminal ganglia (V) and facial and acoustic nerves (VII/VIII), glossopharyngeal nerve (IX), vagus nerve (X) and the spinal accessory nerve (XI) (**d**). **g**, **h** e10.5 mutant mouse lost *Sox10* expression, especially in the facial prominences (**g**, red arrow) and showed remarkably reduced expression in cranial ganglia and nerves X, XI. The dorsal root ganglia seem to be unaffected. **i** *Twist* expression marks migratory neural crest. BIO treatment from st12.5 results in a loss of *twist* expression at st17 (frontal views, st17). BIO treatment from st12.5 to st19 shows loss of twist expression at st20 and st26 (lateral views). The posterior streams are selectively impaired, red arrows (*n* ≥ 35). **j** BIO treatment resulted in a reduction of Alcian blue-stained facial cartilages, which are derived from neural crest (*n* = 12). **k** *Twist* expression shows that cell migration is regained by stage 28, following washout from the treatment with BIO from st12.5 to st19 (*n* = 3). **l** Frontal view of a tadpole at stage 45, previously treated with BIO (st12.5–st19). Note narrowing of the head structures and loss of the mouth (marked with red lines in control). **m**–**p** GFP-labelled neural crest was grafted into a non-labelled embryo at stage 17 and grown to st28. GFP-labelled cells in control animals have migrated (**n**), while those treated with 15 µM BIO have not migrated (**o**, **p**) (*n* = 3)

We then turned to mouse mutants to determine the in vivo genetic requirements for *GSK3* in the neural crest. To do this, we generated embryos with a conditional deletion of both *GSK3α* and *GSK3β* genes using a neural crest-specific cre-recombinase driver (*Wnt1::cre*^26^). By e9.5, we found widening of the NP (Fig. 2a, e, red bracket), with a medial expansion of *Sox10* expression (Fig. 2a, e, asterisk). By e10.5, dorsal views revealed an accumulation of *Sox10*-positive cells in the brain (compare Fig. 2b, f, red bracket), suggesting that the cranial neural crest cells have failed to migrate from the neural tube. Complete nulls had a severe disruption of cell migration to the facial prominences, the branchial arches and the cranial ganglia (compare *Sox10*-positive/blue in Fig. 2c, d to 2g, h). Because we rarely found animals surviving beyond e11.5, we also examined animals carrying a single allele of functional GSK3 (*Wnt1::cre; GSK3α*^*fl/+*^*;β*^*fl/fl*^ or *Wnt1::cre; GSK3α*^*fl/fl*^*;β*^*fl/+*^). In both cases, we noted a reduction in *Sox10*-positive cells en route to the first branchial arch and an accumulation of positive staining in the neural tube, suggesting that both GSK3 proteins contribute to migration of the neural crest (Supplementary Figure 1A,C,E,G red bracket and B,D,F,H dotted square).

While the timing of the *Wnt1::cre* transgene misses the initiation of neural crest induction, it was still possible that some of the effects seen were due to GSK3 requirements in the pNC. In order to bypass early effects on the neural crest, we turned to pharmacological inhibition of GSK3 using the specific inhibitor BIO (6-bromoindirubin-3′-oxime)^27^. Making use of *Xenopus* allowed us to precisely time our manipulations in a well-defined in vivo system. Treatments of *Xenopus* embryos confirmed that GSK3 inhibition led to loss of the migration marker *twist1* (Fig. 2i). Treatment of *Xenopus* embryos at stage 12.5 confirmed that GSK3 inhibition led to loss of *twist1*. When the embryos were released from treatment at stage 19, we found some recovery of *twist1* expression (Fig. 2k). Loss of GSK3 function during the critical stages led to significant changes in face shape as well as a smaller neural crest-derived tail fin (Supplementary Figure 2A-C), as well as loss of the neural crest-derived facial cartilages (Fig. 2j, l). Although the facial cartilages were lost leading to narrowing of the head, the mesodermal cranial muscles are still formed (Supplementary Figure 2D). To confirm that this effect was specifically due to impairment of migration, we transplanted fluorescently labelled neural crest cells into a stage 17 embryo and then treated with BIO; these cells did not migrate (Fig. 2m–p). Taken together, this demonstrated a previously under-appreciated role for GSK3 during neural crest cell migration.

### GSK3 activity favours migration of cranial neural crest

**Fig. 3:**
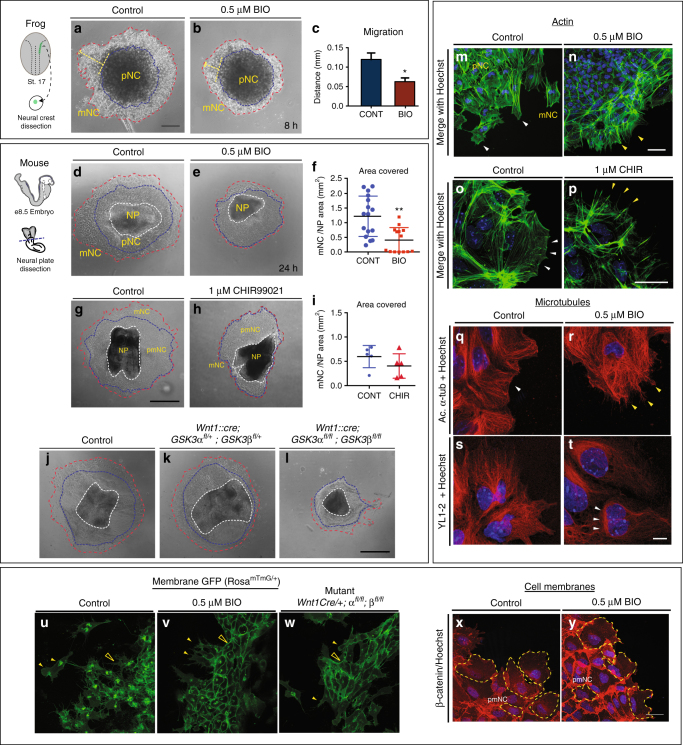
GSK3 activity is required for neural crest cell migration. **a**, **b** Neural crest cells from st 17 *Xenopus* embryos (*n* = 9). **a** Control explants after 8 h in culture. **b** Upon GSK3 inhibition, neural crest cells spread significantly less than the controls. **c** Quantification of the distance migrated *p < 0.05. (**d**, **g**, **j**) Control mouse explants. **e**,** h** Explants treated with 0.5 µm BIO or 1 µM CHIR99021, respectively. Note the decrease in area covered by migratory neural crest (**f**, refers to **d**, **e**; **i** refers to **g**, **h**). Each dot represents one explant (**p < 0.001, unpaired *t*-test) **j**–**l** Mouse explants from control (**j**), *Wnt1::cre; GSK3afl/+; GSK3bfl/*+ (**k**) and *Wnt1::cre; GSK3afl/fl; GSK3bfl/fl* complete mutants (**l**). Note the decrease in area covered in **l** (red dotted line). **m**–**p** Filamentous actin (green) in neural crest explants. **m**,** o** Explants treated with DMSO (control) show accumulation of F-actin in lamellipodia in the leading edge (white arrowheads). Explants treated with **n** 0.5 µm BIO and **p** 1 μM CHIR lack lamellipodia and only show stress fibres at the cell edge (yellow arrowheads). **q**–**t** Microtubules are labelled with acetylated α-tubulin or YL1-2. Controls show smooth lamellipodial edge (**q**) but BIO-treated cells show spiky protrusions (**r**). YL1-2 tubulin in control cells is distributed all throughout the cell (**s**); in BIO-treated cells (**t**), it is found in a perinuclear zone, at the rear of the nucleus. **u** Cranial neural crest explants from control e8.5 embryos carrying membrane GFP in the neural crest lineage. Migrating cells show an elongated morphology and have a dynamic cell–cell contact (see Supplementary Movies 1, 3, 6). Membrane GFP is unstable and intracellular. **v** In the BIO treatment, cells remain in contact with adjacent cells and multiple protrusions (yellow arrows). **w** Mutant cells carrying a neural crest-specific deletion of GSK3 (*Wnt1Cre/*+; *GSK3afl/fl; GSK3bfl/fl*) lose motility and maintain stable cell–cell contacts and membrane GFP. **x**, **y** β-Catenin staining in neural crest explants. β-Catenin is stable in BIO-treated cells (**y**). **a**–**l** Scale bar = 100 µm. **m**–**y** Scale bar = 25 µM. All are representative images from at least three independent experiments, except from **j** to **l** and **w** in which only one of each genotype was obtained

To bypass the neural crest induction stage as well as the embryonic lethality, we turned back to the neural crest explant cultures. We found that dispersion of *Xenopus* cells was inhibited by BIO treatment (Fig. 3a–c). Note that, in the *Xenopus* explants, the pNC population can be specifically dissected independently from the NP. We then turned back to mouse neural crest cultures in order to better compare our pharmacological inhibitors to genetic mutants. Treatment with two different specific inhibitors, BIO and CHIR99021 (CHIR^28^) prevented neural crest cell migration similar to that observed in *Xenopus* (Fig. 3d–i, Supplementary Movies 1, 2). We found that the area covered by the pNC cells was expanded in treated samples (Supplementary Figure 3A, 3B) with a concurrent decrease in the migratory population (Supplementary Figure 3B), suggesting a defect or delay in the cells emigrating from the leading edge of the neuroepithelium. This was confirmed in genetic mutants, where a complete loss of GSK3 (*Wnt1::cre; GSK3α*^*fl/fl*^*;β*^*fl/fl*^) also led to a decrease in mNC cells (Fig. 3j–l).

One possibility was that inhibitory serine phosphorylation of GSK3 is necessary at the leading edge of polarized cells as has been demonstrated in astrocytes and neurons^29–32^. However, mouse mutants carrying non-phosphorylatable S21A/S9A substitutions (*GSK3α*^*S21A/S21A*^*; GSK3β*^*S9A/S9A*^) are viable, suggesting normal neural crest migration^11^. Nevertheless, to confirm this, we observed that neural crest explants from these mice appear normal and retain sensitivity to BIO inhibition, demonstrating that serine phosphorylation of GSK3 is dispensable during neural crest migration (Supplementary Figure 4A,B). Therefore, because of the polarized expression of pY-GSK3 at the leading edge, we decided to focus on the role of GSK3 as the neural crest cells acquire their mesenchymal nature.

### GSK3 inhibition perturbs the cytoskeleton

GSK3 activity has been predicted to control cytoskeletal dynamics in a number of systems. The loss of cell migration in mutant or BIO-treated cultures suggested a disruption of cytoskeletal dynamics; therefore, we examined the actin and microtubule arrangements in neural crest cells after inhibition of GSK3 (Fig. 3m–t). GSK3 inhibition markedly increased stress fibres and concurrently reduced leading-edge actin (Fig. 3m–p, Supplementary Figure 5A). Cell shapes at the leading edge were markedly different, with both BIO- and CHIR-treated cells losing filamentous actin localization, which is normally at the edge of the cell (see Fig. 3m, o, white arrowheads) and generating spiky protrusions (Fig. 3n, p, yellow arrowheads). Microtubule organization was also disrupted in BIO-treated cells. In normal cells, stabilized microtubules (marked by acetylated tubulin) extend from the centrosome toward the leading edge of the cell (see Fig. 3q). In BIO-treated cells, stabilized microtubules appeared to accumulate perinuclearly (Fig. 3r, Supplementary Figure 5C). We also examined a marker for unstable microtubules (YL1-2^33^) and found a significant decrease in this population, which also accumulated primarily at the rear of the cell, behind the nucleus (Fig. 3s–t, arrowheads and Supplementary Figure 5D). Finally, we examined membrane dynamics in both mutants and BIO-treated explants from mice carrying a genetically labelled membrane green fluorescent protein (GFP) (Fig. 3u–w). In the control explants, a large proportion of the GFP was internalized within the cell, suggesting recycling of the membrane (Fig. 3u, closed arrowhead). Instead, both treated and mutant cells had very strong expression of GFP at the cell membrane (Fig. 3v, w, open arrowheads). Consistent with these findings, we also found an accumulation of β-catenin at the membrane in BIO-treated explants (Fig. 3x, y), suggesting that loss of GSK3 activity led to extremely stable membrane dynamics compared to controls.

**Fig. 4:**
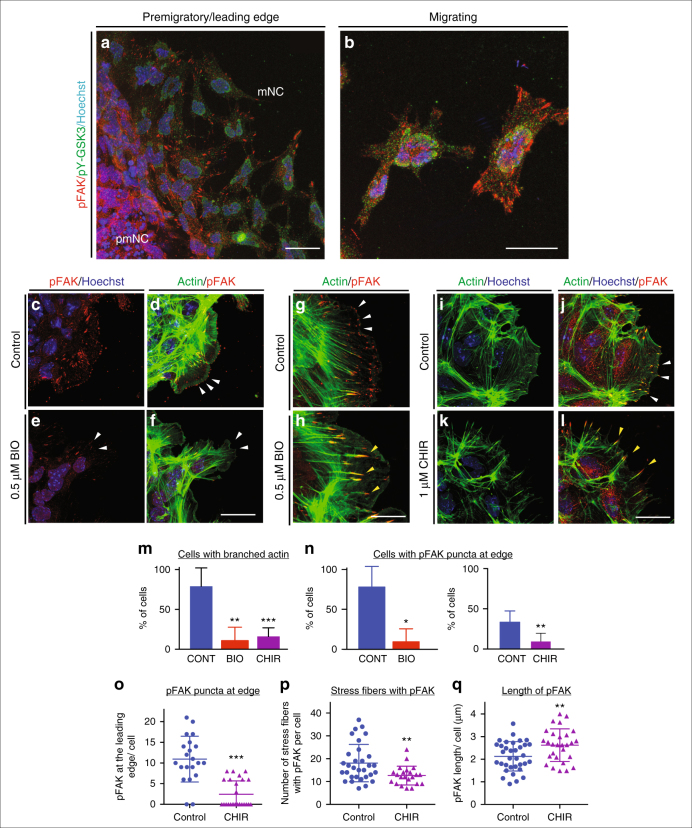
GSK3 allows FAK localization to establish lamellipodial protrusions at the leading edge in migrating neural crest cells. **a**,** b** Immunofluorescence for pFAK-Y397 (pFAK, red) and pY-GSK3 (green). **a** In control explants, pFAK is found at the leading edge of the delaminating cells and in migrating cells. **b** In migratory neural crest cells, pYGSK3 and pFAK are mutually exclusive. **c**, **d** pFAK is found in puncta at the leading edge of the cell co-localizing with lamellipodia. **e**,** f** Upon GSK3 inhibition, the cells lose pFAK at the leading edge. **g**, **h** pFAK accumulates at the tips of actin-rich fibres. Note the increase in length of pFAK associated with actin upon treatment with BIO (**h**). **i**,** j** Similarly, treatment with 1 µM CHIR elicits the same response (white and yellow arrowheads). **a**–**l** Scale bar = 25 µM. **m**,** n** Percentage bar charts representing a significant decrease in cells with branched actin (**m**) or showing pFAK puncta at the leading edge (**n**) when GSK3 is inhibited with either BIO or CHIR; **p ≤ 0.001 and ***p ≤ 0.0001, two-tailed unpaired *t*-test. **o**–**q** Dot plots representing the number of pFAK puncta at the edge (**o**), the number of stress fibres containing pFAK (**p**) and the length of of pFAK (**q**) in control and CHIR-treated cells; each dot represents one cell. **p ≤ 0.001 and ***p ≤ 0.0001, two-tailed unpaired *t*-test

**Fig. 5:**
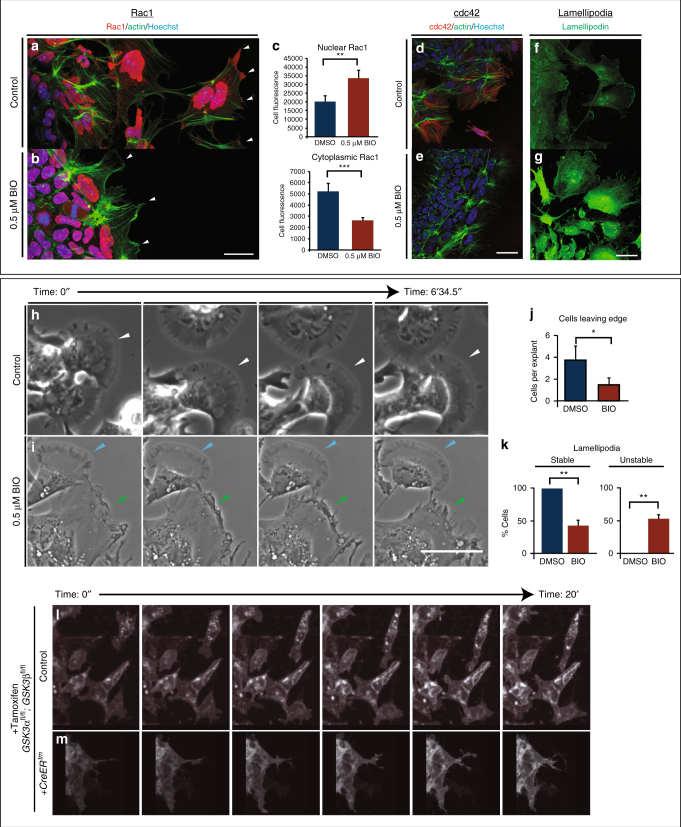
GSK3 is required to establish polarity and to form lamellipodia in migrating neural crest cells. **a**, **b** GSK3 inhibition increases nuclear RAC1 and reduces cytoplasmic RAC1 in neural crest cells. **a** In control explants, RAC1 (red) is high in the nucleus while in the cytoplasm it is enriched at cell protrusions (arrowheads). Actin was labelled with phalloidin (green). **b** In BIO-treated explants, nuclear RAC1 is high but cytoplasmic staining is lost (white arrowheads). **c** Relative levels of Rac1 fluorescence in the nucleus or the cytoplasm (*n* = 9) **p ≤ 0.001 and ***p ≤ 0.0001, two-tailed unpaired *t*-test. **d**–**e** GSK3 inhibition reduces the expression of cdc42 in neural crest cells. **d** Cdc42 is cytoplasmic and perinuclear in neural crest cells. **e** BIO-treated explants lose cdc42 staining. **f**–**g** In controls, anti-lamellipodin (green) stains the ruffled edge of migrating cells. **g** In contrast, BIO-treated cells show increased total lamellipodin throughout the cell, losing specific localization at the cell edge. Scale bar, 20 μm. **h**,** i** Bright-field still images from controls (**h**) or BIO-treated samples (**i**) (*n* = 3 explants with ≥10 cells per explant). Control images show cells form fan-shaped stable lamellipodia and ready to migrate away from the cluster (**h**, white arrowheads). In treated cells, despite some cells form stable lamellipodia as found in controls (**i**, light blue arrowheads), cells predominantly formed an irregular protrusion that tend to retract (**i**, green arrowheads). Scale bar, 500 μm (see corresponding Supplementary Movies 6, 7). **j** Graph showing the number of cells delaminating from the premigratory neural crest cell clusters in 2 h. *p ≤ 0.05 and **p ≤ 0.001, two-tailed unpaired *t*-test. **k** Percentage of delaminating cells that show stable (persistent) lamellipodia or unstable (short-lived) lamellipodia. **l**, **m** Still images of time-lapse showing control mouse neural crest cells (**l**, supplementary movies 3,5) and *pCAAG::creER*^*tm*^ *;GSK3α*^*fl/fl*^ *; GSK3β*^*fl/fl*^; *Rosa*^*mtmg/+*^ deleted cells (**m**, supplementary movie 4, 5). Upon tamoxifen-induced knockout of GSK3, the neural crest cells are unable to migrate and the cell edge does not form lamellipodia

One candidate GSK-3 substrate is focal adhesion kinase (FAK), a well-known regulator of cell motility, which controls focal adhesion dynamics and turnover required for formation of branched actin (as opposed to linear actin). In motile cells, FAK is regulated by an activating tyrosine phosphorylation and a series of inhibitory serine phosphorylations, which are mutually exclusive; of these, S722 is a known GSK3 target^34,35^. Consistent with GSK3 roles in inhibiting FAK, we found that pY-GSK3 (in green) and pY-FAK (in red) were mutually exclusive in the cytoplasm (Fig. 4a, b). In addition, we found that mNC cells ordinarily express active pY-FAK in a halo of puncta that are oriented towards the direction of cell movement, which is towards the right side of all figures (Fig. 4c, d, g, j). This punctate expression is reminiscent of microspikes or transient actin-filled filopods, which initiate actin nucleation filaments. The percentage of cells with a loss of branched actin (Fig. 4l–m) and containing pFAK at the edge was consistently reduced after GSK3 inhibition (Fig. 4f, n, o), suggesting a loss of lamellipodia-like characteristics. Instead, loss of GSK3 led to a striking accumulation of active pY397-FAK in long extensions indicating persistent focal adhesions (Fig. 4h, p–q). FAK is thought to control cytoskeletal dynamics by repressing the function of the small GTPase Rac1^36^. Therefore, the inhibition of FAK activity appears necessary to allow Rac1 activation. With the accumulation of active FAK in our treated cells, we found that Rac1 was now excluded from the leading edge of the mNC cells (compare Fig. 5a to Fig. 5b, arrowheads). Interestingly, we see an increase in Rac1 in nuclei (Fig. 5b, c), which could indicate a more direct role for GSK3 in subcellular localization of Rac1, possibly via phosphorylation of the Rac1 regulator nucleophosmin/B23^37,38^. We saw a concurrent loss of cdc42 localization to the leading edge of the cell (Fig. 5d, e). Finally, since FAK and Rac1 can control lamellipodial movement, we examined the localization of lamellipodin, which regulates neural crest migration via the actin effectors Ena/VASP and Scar/WAVE^39,40^. When GSK3 is inhibited, leading-edge localization of lamellipodin is lost, and it also relocalizes to the nucleus (Fig. 5f, g). As a consequence, treated neural crest cells fail to generate stable fan-shaped lamellipodia (Fig. 5h–k, green arrowheads), with approximately 50% of delaminated cells having unstable lamellipodia (Fig. 5k). We then turned back to genetic mutants to confirm these phenotypes (Fig. 5l–m). In this case, to bypass any complications of GSK3 involvement in neural crest induction, we turned to a tamoxifen-inducible Cre (*pCAAG::CreER*^*TM*^)^41^, allowing temporal deletions upon drug addition. As predicted, tamoxifen-induced knockout of *GSK3* led to a loss of the wave-like lamellipodial protrusions, leaving only spiky filopodial movements in neural crest cells (stills shown in Fig. 5l–m and Supplementary Movies 3–5). All together, this demonstrates that in the absence of GSK3 activity, neural crest cells make filopodial protrusions at the expense of lamellipodia.

### ALK is expressed in mNC cells

**Fig. 6:**
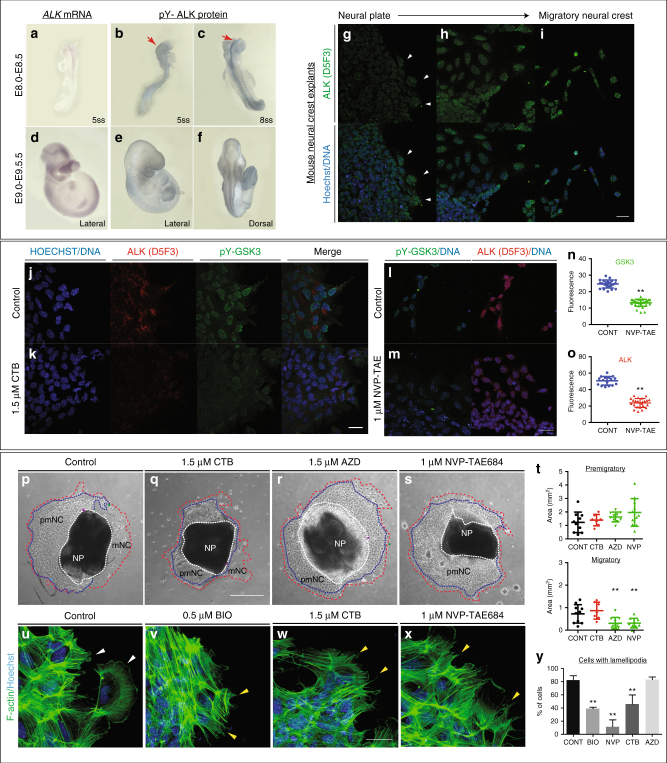
Inhibition of ALK leads to decreased levels of pY-GSK3 in mouse neural crest. **a**–**f** ALK is expressed in the neural crest. (**a**, **d**) mRNA in situ hybridization for *Alk* in e8.5 and e9.5 mouse embryos. **b**,** c**, **e**, **f** Antibody staining for activated ALK protein shows expression at the neural plate border (red arrows) and in the branchial arches. **g** Staining for total ALK (green). **g** Very little total ALK is present in the neural plate with some present at the edge of the premigratory neural crest (white arrowheads). **h** Migratory neural crest cells express higher levels of ALK. **i** In fully migratory NC cells, ALK appears to be nuclear. Underneath each panel, ALK merged with Hoechst. **j** Co-immunostaining of pY-GSK3 (green) and ALK (red) show that ALK is expressed in all cells that express pY-GSK3. **k** Treatment with the ALK inhibitor crizotinib (CTB) for 24 h reduces the levels of pY-GSK3. **l**,** m**, **n**,** o** Quantitation of loss of pY-GSK3 and ALK fluorescence using the alternative inhibitor NVP-TAE also results in a loss of mean fluorescence intensity. **p**–**s** Bright-field images of neural crest explants treated with vehicle control or three different ALK inhibitors: 1.5 µM CTB, 1.5 µM AZD-3463, or 1 µM NVP-TAE-684. All three treatments showed a loss of migratory neural crest (red dotted lines) (*n* = 3 explants with ≥10 cells per explant, scale bar = 500 µm). **t** Area quantification of the premigratory neural crest (pNC, area depicted by blue dotted lines covered in **p**–**s**) and the migratory neural crest population (mNC, red dotted lines). A significant reduction in mNC was seen in AZD and NVP treated explants. **u**–**y** Phalloidin staining shows F-actin structure in explants. Hoechst marks nuclei. Note the loss of lamellipodial structures upon ALK inhibition (**w**, **x**) is comparable to BIO treatment (**v**) (scale bar = 25 µm). **y** Percentage of cells with lamellipodial formations at the leading edge upon treatment with GSK3 inhibitor BIO or with ALK inhibitors (*n* = 10–20 cells per explant). Bars, mean ± SD. One-way ANOVA and unpaired *t*-test *P < 0.05, **P < 0.01

Aberrant neural crest development is thought to underlie NB, an aggressive paediatric cancer. Activating mutations in ALK contribute to a subset of NB cases, correlating with poor prognosis^42–47^. Because we saw specific expression of pY-GSK3 at the leading edge and in mNC cells, we wondered whether ALK might be responsible for activating GSK3. First, we set out to check whether ALK was expressed during the appropriate stages of neural crest development. Expression of ALK has previously been reported at e10.5, including in the diencephalon and facial ganglia^48^; however, to our knowledge, there has been no analysis performed during key neural crest migration stages. To test this, we performed mRNA in situ hybridization from e8.0 onwards (Fig. 6a, d). We found that, by e8.5, ALK appeared specific to the NP border (NPB) corresponding to active cranial crest migration (Fig. 6a). This expression was enriched at the NPB consistent with a role for ALK in the delaminating neural crest cells (Fig. 6a, d). Furthermore, ALK continues to be expressed at 9.5 days post coitum (dpc) at the NPB and in a mNC destined for branchial arch I and II and at the frontonasal process (Fig. 6d). Additional expression was seen in the heart, trunk and limbs. We also examined localization of the active form of ALK protein. Using an antibody recognizing ALK carrying a phospho-tyrosine residue (pY1507-ALK), we again found enrichment of ALK in the right place at the right time to be acting upon GSK3 during neural crest migration (Fig. 6b–f). This neural crest-specific expression was recapitulated in explant cultures, where we noted a lack of ALK protein in NPs (total ALK, Fig. 6g) followed by an onset of expression in mNC cells, which was somewhat nuclear, with diffuse staining throughout the cells (compare Fig. 6h–i).

### Inhibition of ALK activity leads to a loss of pY-GSK3

To test whether ALK activity is required for tyrosine phosphorylation of GSK3, we challenged the neural crest explants with specific inhibitors for ALK. These included crizotinib (CTB), which is currently used as a chemotherapeutic, and AZD3463 (AZD). Because both of these are dual function inhibitors (CTB-blocking ALK and the c-met receptor^49,50^, and AZD-blocking ALK and insulin-like growth factor receptors^51^), we also treated with the highly selective inhibitor NVP-TAE684 (NVP^52^) (Fig. 6l, m). We found that blocking ALK led to a loss of pY-GSK3 expression in neural crest explants, suggesting that GSK3 was indeed a target of ALK kinase activity in this context (Fig. 6k, m, n). Furthermore, in all three cases, blocking ALK function phenocopied loss of GSK3 activity leading to a substantial decrease in delamination of the neural crest and a loss of the migratory cell population (Fig. 6p–s). We noted that NVP treatment was the most effective at blocking neural crest migration while CTB had a much milder effect (Fig. 6s). Finally, ALK inhibitors CTB and NVP phenocopied the disruption of the actin cytoskeleton seen when GSK3 was blocked (Fig. 6u–y).

### NB with high levels of ALK co-express pY-GSK3

**Fig. 7:**
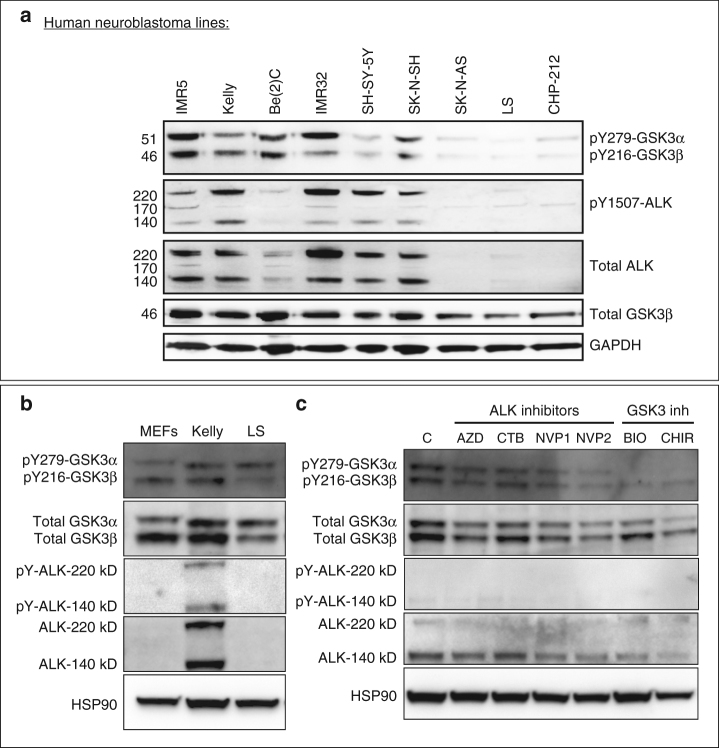
Neuroblastoma lines with high levels of active ALK also have high levels of pY-GSK3. **a** Western blotting of neuroblastoma lines reveals levels of pY-GSK3 correlates with levels of ALK-pY1507 (IMR5, Kelly, Be(2)C, IMR32, SH-SY-5Y, SK-N-SH). Cell lines with low or no ALK-pY1507 (SK-N-AS, LS, CHP-212) have correspondingly low levels of pY-GSK3. **b** Western blotting showing that only Kelly cells have high levels of ALK when compared to mouse embryonic fibroblasts (MEFs) and LS neuroblastoma cells. **c** Western blotting analysis reveals that, in Kelly cell line, ALK inhibition with NVP-TAE, results in gradual loss pYGSK-3α and pYGSK-3β, are significantly reduced after 24 h treatment with NVP-TAE, GSK3 inhibition, using BIO or CHIR, leads to a reduced expression of pY-GSK3, more predominantly is pY-GSK3α isoform. Some loss of ALK is also seen in NVP treatments. Treatments used were 1.5 µM crizotinib, 1.5 µM AZD-3463, 1 µM NVP-TAE-684 (NVP1), 2 µM NVP-TAE-684 (NVP2), 0.5 μM BIO and 1.0 μM CHIR99021

We then wondered whether high levels of ALK activity could drive excessive activation of GSK3. To test this, we examined nine NB lines and found a clear correlation between levels of total ALK, active (pY-1507) ALK and pY-GSK3 (Fig. 7a, Supplementary Figures 6, 7). We then focussed on the Kelly NB line, which carries an activating mutation in ALK (F1174L)^53^ and, as a comparison, LS^54^ NB cells which had very low levels of ALK. In western blots, we again found much higher levels of ALK in Kelly cells, with nearly undetectable levels in LS cells, more similar to that of mouse embryonic fibroblasts (MEFs) (Fig. 7b, Supplementary Figure 8).

### ALK activity is required for pY-GSK3 in NB lines

All together, our data raised the possibility that ALK regulation of GSK3 is a neural crest-specific activity that may have been co-opted during cancer progression. Indeed, inhibition of ALK in the NB lines also decreased pY-GSK3 levels (Fig. 7c, Supplementary Figure 9). The Kelly NB line carries an ALK-F1174L mutation, which renders it somewhat insensitive to the ALK inhibitor CTB^53^. Therefore, as with the neural crest explants, we confirmed these findings using the two other inhibitors AZD and NVP (Fig. 7c, Supplementary Figure 9). We found that treatment with ALK inhibitors was sufficient to decrease phosphorylation of GSK3 (Fig. 7c, Supplementary Figure 9B, E). Treatment with BIO or CHIR also affected pY-GSK3 levels, consistent with some auto-regulation by GSK3 itself (Fig. 7c).

**Fig. 8:**
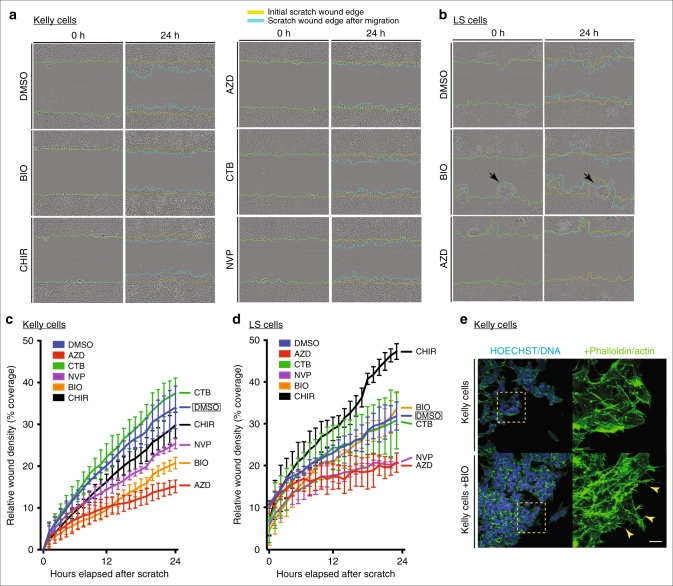
GSK3 and ALK inhibition affect migration in neuroblastoma cell lines. **a**, **c** Cell migration assay for Kelly neuroblastoma cell line. **a** Representative bright-field still images at start (*t* = 0 h) and end (*t* = 24 h) time points of the migration assay in Kelly cells under various GSK3 (0.5 μM BIO, 1.0 μM CHIR99021) and ALK inhibition treatments (1.5 μM CTB, 1.5 μM AZD-3463 and 1.0 μM NVP-TAE684). **c** Line graph representing the percentage of wound coverage over time. Notice that upon NVP-TAE684 (NVP) and BIO treatments cells do not close the wound as quickly as the control or unaffected CTB samples. AZD treatment showed the lowest percentage of wound coverage. **b**, **d** Cell migration assay for LS neuroblastoma cell line. **b** Representative bright-field still images at start (*t* = 0 h) and end time (*t* = 24 h) points of the migration assay in LS cells under GSK3 inhibition (0.5 μM BIO) and ALK inhibition treatment (1.5 μM AZD-3463). Note that upon BIO treatment LS cells tend to aggregate and expand into the wound (black arrows). **d** Line graph representing the percentage of wound coverage in LS cells. There is a tendency to increase migration upon GSK3 inhibition (BIO and CHIR). The lower wound coverage upon ALK inhibition treatment correlates with reduced population of cells at the end of the assay suggesting a compromise in cell viability under these conditions. **e** Representative images of actin staining (phalloidin, green) showing Kelly cells treated with BIO compared to controls. Notice the irregular spiky protrusions of cells where GSK3 is inhibited (yellow arrowheads). Scale bar, 25 μm

Finally, we set out to determine whether the excessive levels of pY-GSK3 could underlie the aggressive nature of NBs. If GSK3 activity is downstream of ALK in this context, we would predict that inhibition of GSK3 in Kelly cells would be sufficient to limit cell migration. Indeed, using scratch assays where we measured the movement of cells, we observed that Kelly cell migration was blocked by ALK inhibitors (NVP/AZD) similarly to GSK3 inhibition (BIO/CHIR) (Fig. 8a, c). As noted before, Kelly cells were resistant to CTB (Fig. 8a, c).

In contrast to the Kelly cells, LS cells, which do not carry the ALK-F1174L variant, behaved very differently. LS cells had substantially less pY-GSK3, which correlated with much lower levels of active ALK (pY1507, Fig. 7a or pY1586, Supplementary Figure 6A). LS cells were insensitive to CTB treatment (Fig. 8b, d and Supplementary Figure 6B–D). But, while the other ALK inhibitors led to a substantial decrease in the area covered, examination of the cultures showed substantial cell death (see Fig. 8b, bottom right panel). More interesting, we found that GSK3 inhibition in LS cells elicited an unusual phenotype in the scratch assays, with cells moving together in aggregates, rather than as single cells (Fig. 8b, arrowheads). Our interpretation is that NB lines are developmentally heterogenous. Therefore, some, like LS cells, may represent neural crest cells that have a more 'epithelial' morphology, while Kelly cells represent neural crest cells that have fully progressed through EMT. Depending on the developmental status of the NB line, the impact of GSK3 loss will be different (e.g., acting via EMT versus acting via the actin cytoskeleton and cell motility). Consistent with our hypotheses, morphologically, the Kelly cells responded similarly to motile neural crest cells, with BIO-treated Kelly cells appearing compacted with dense actin staining (Fig. 8e). Nevertheless, taken together, our data suggest that ALK activity is closely linked to GSK3 phosphorylation and activity in primary neural crest and NB cells.

## Discussion

A key defining feature of the neural crest lineage is the ability to undergo EMT, acquire motility, migrate and, ultimately, to differentiate into diverse cell types during embryonic development. Aberrant neural crest development results in neurocristopathies and other malignancies such as cancer. Thus the same migratory characteristics could contribute to tumorigenesis and metastasis. The understanding of cellular behaviours in the normal context can aid our identification of important molecules involved in abnormal cell behaviours.

Here we studied the effect of the serine/threonine kinase GSK3 during mammalian neural crest migration. Previously, we have found that GSK3β is required for the palate formation at specific time points during development^6^. This structure depends upon neural crest migration. However, due to functional redundancy between GSK3α and GSK3β, the in vivo activity has been difficult to study^55^. Furthermore, early loss of GSK3 leads to global Wnt activation, which can obscure later developmental roles^22,23,56–58^. However, our studies bypass these early complications and provide a refined understanding of the regulation and function of GSK3. This effect on neural crest migration appears to be β-catenin independent, as neural crest-specific expression of stabilized β-catenin does not disrupt migration^59^. Instead, GSK3 appears to act directly on the actin cytoskeleton, changing the dynamics of lamellipodial formation. Our data demonstrate that this regulation may be via regulation of FAK localization, as well as key downstream factors, including Rac1, cdc42 and lamellipodin. Interestingly, neural crest-specific deletion of *FAK*, *Rac1*, *cdc42* and *lpd* all have a range of craniofacial anomalies^60,61^. However, it is worth noting that, in our assays, we predominantly found that these proteins were relocalized, and thus it is difficult to directly compare our observations with the published null phenotypes. Nevertheless, these observations are worth further in-depth study.

GSK3 can also regulate the dynamics of the actin cytoskeleton, microtubules and cell-to-matrix adhesions^62^. To date, polarized inhibition via phosphorylation of S9 of GSK3β has been thought to be the main mechanism for establishment of cell polarity, especially in astrocytes^30^, and is also critical for glioma cell invasion^63^. All of these scenarios depend on negative regulation of GSK3. Importantly, contrary to the neuronal cell scenario, we find that GSK3 inhibition via serine phosphorylation is not necessary for neural crest migration (Supplementary Figure 4). However, given the complexity of GSK3 regulation, it would be interesting to see whether combining phosphorylation mutations on the activating tyrosines and inhibitory serines has an additive effect on neural crest migration.

Most important, we find that neural crest cell migration depends on GSK3 activity and that this correlates with high levels of tyrosine phosphorylation via ALK. While there are other kinases that may be regulating GSK3 phosphorylation, including GSK3 itself^16^, the association with ALK in the context of NB is particularly compelling. However, future studies should include other tyrosine kinases that may be phosphorylating GSK3. For instance, it has been reported that PYK2^64^, a putative mammalian homologue of ZAK1, is a kinase found in *Dictyostelium* shown to phosphorylate GSK3β at Y216. However, there is still no clear evidence on how this finding could relate to mammals. In pathological conditions, pY216-GSK3β has been found in prostate cancer, and Src was found to promote this phosphorylation, and with it, cancer progression and invasion^65^. These other kinases are worth considering in the future and may be necessary to regulate sub-populations of GSK3.

Particularly intriguing was the prediction that GSK3α is a putative ALK substrate in cancer cells^18^. ALK is negatively associated with NB prognosis, with hyperactivating mutations of this kinase found in some of these aggressive tumours. Therefore, the discovery that pY-GSK3 was specifically expressed in delaminating and mNC cells and that this correlated with ALK-positive NB cells was extremely exciting. The additional discovery that ALK inhibition perturbs neural crest migration as well as the expression of pY-GSK3 provides strong evidence for a new signalling cascade regulating neural crest cellular behaviour. Finally, because we focussed on cell motility and lamellipodia formation, we cannot exclude the possibility that the ALK-GSK3 pathway has additional or longer-term effects on neural crest differentiation. NB lines carrying ALK-F1174L have also has been suggested to regulate serine (S9) phosphorylation of GSK3β via activation of the PI3K/AKT pathway. This leads to the stabilization of MYCN resulting in the formation of aggressive, highly penetrant tumours^43^. Because this serine phosphorylation was not required in the endogenous neural crest, we did not address the status of inhibited (phospho-serine) GSK3 in NB cells. Nevertheless, this raises the possibility that phosphorylation events are necessary to set up distinct pools of GSK3 within the cell, which then regulate cell motility, transcriptional activity or protein localization.

All together, our study demonstrates that the timing and control of active GSK3 within the embryo plays important roles during multiple steps in neural crest development. These lessons from the embryo improve our understanding of the pathological misregulation of key kinases, in NB and congenital anomalies, and may have broader implications for cell motility in diverse systems.

## Methods

### Animal procedures

All animal work was performed at King’s College London in accordance with UK Home Office Project Licenses 70/7441 and P8D5E2773 (KJL). CD-1 mice were obtained from Charles River Laboratories. Mouse lines have all been described previously and are summarized in Supplementary Table 1. In brief, for deletion of *GSK3*, we used: *GSK3α*^*lacZ*^, *GSK3β*^*lacZ*^, *GSK3α*^*fl*^, and *GSK3β*^*fl*^ mouse lines. *pCAGG::Cre-ER*^*T2*^ and *Wnt1::cre; Tg(Wnt1-cre)11Rth* Cre drivers were used and *R26R*^*mTmG*^ as a reporter line. Genotyping was performed as described in original publications. All mouse lines were backcrossed to CD1 to maintain consistency in the background strain used. The gestational ages were determined based on the observation of vaginal plugs, which was considered e0.5. Embryos were further staged by counting the number of somites after dissection. For each experiment, a minimum of three mutants with litter-matched controls were studied unless otherwise noted. Sex of embryos was not determined. *Xenopus laevis* embryos were cultured using standard methods^66^.

### Mammalian cell culture

MEFs were prepared according to standard procedures and cultured in Dulbecco’s modified Eagle’s medium (Sigma) supplemented with 10% (v/v) foetal bovine serum (FBS), 2mM L-glutamine, Pen/Strep, 15 mM HEPES and b-mercaptoethanol (all from Invitrogen). NB cell lines LS and Kelly were cultured in RPMI media supplemented with 10% (v/v) FBS and Pen/Strep. For tamoxifen-dependent cre deletions in culture, 4-OH-tamoxifen (Sigma H7904 5 mg) was applied at 2 μg/ml for 24 h. After the incubation period, the media was replaced with standard media and cultured at 37 °C and 5.0% CO_2_.

Mouse primary cranial neural crest cultures were performed according to established protocols^67,68^. For mouse cranial neural crest explants, dissections were performed on embryos at 8.5 dpc and only embryos at the 5–8 somite stages were used. Briefly, the embryo was positioned to visualize and excise the neural fold. Adjacent tissues, such as mesoderm, were carefully cleaned from the NP. The NP was then cultured on matrigel-coated plates or slides in basal neural crest media at 37 °C, 5.0% CO_2_ for 24 h to allow migration of neural crest cells out of the NP. When drug treatment was applied, it was added at plating (*T* = 0).

NB cell lines used are noted in Supplementary Table 2 and Supplementary References. All cell lines were mycoplasma tested.

### Antibodies

For the list of primary and secondary antibodies used for immunofluorescence and western blotting, please refer to Supplementary Table 3.

### Immunoblotting

Cells were washed twice with phosphate-buffered saline (PBS) and lysed with RIPA lysis buffer with added phosphatase inhibitor (PhosStop, Roche) and protease inhibitor (cOmplete^TM^, Roche). Protein concentrations were determined by Bradford protein assay (BioRad). Proteins were resolved by sodium dodecyl sulfate-polyacrylamide gel electrophoresis on 4–12% precast gels (NuPAGE®Bis-Tris), then transferred to polyvinylidene difluoride membrane (IPVH00010, Millipore) using Trans-Blot® SD semi-dry transfer cell (BioRad). Immunoblots were developed using chemiluminescent horseradish peroxidase substrate (ECL) (Immobilon, Millipore) and ChemiDoc^TM^ Touch Imaging System (BioRad) for detection. Densitometry was performed using the Fiji-ImageJ analysis software, and all the values were normalized to HSP-90 or GAPDH controls per standard approaches. Uncropped blots can be found in Supplementary Figures 7–9.

### Embryo fixation and histology

Mouse embryos at e10.5 were collected in cold PBS and fixed overnight in 4% paraformaldehyde at 4 °C. Mouse embryos at e8.5, e8.75 or e9.5 were collected in cold PBS and fixed in 4% paraformaldehyde for 2 h at 4 °C.

Samples were subsequently washed in PBS three times at room temperature (RT) for 10 min each time, then treated as corresponding subsequent method. For sections, samples were cryoprotected by incubating at 4 °C overnight in graded sucrose solutions in PBS. Tissues were then embedded in Optimal Cutting Temperature and frozen at −20 °C. Heads and bodies were sectioned at 10 μm and frozen at −20 °C after drying at RT for 1 h. For whole mount mRNA in situ hybridization, β-galactosidase activity and immunohistochemistry, samples were treated according to standard procedures.

*Xenopus* embryos were collected at the indicated stages and fixed for 1 h in MEMFA at RT, before washing into ethanol for storage.

*ALK* cDNA clone was obtained from Source Biosystems (ID D130039F03). *Sox10* cDNA was a gift of the Pachnis laboratory^69^. *Life-ActGFP* constructs were a gift of the Mayor laboratory^39^.

### *Xenopus* cartilage staining

Stage 45+ embryos were fixed in MEMFA for 1 h at RT before washing into ethanol. For cartilage staining, embryos were washed into a 0.15% alcian blue solution (70% EtOH/30% acetic acid) at RT for 3 days. When suitably stained, embryos were rinsed 3 × 15 mins in 95% EtOH. Rehydration was done stepwise into 2% KOH, then washed from 2% KOH stepwise into 80% glycerol/20% 2%KOH, 1 h per wash before washing overnight into the final solution. Dissection of cartilages was then performed to increase visibility of craniofacial cartilages.

### Dissection of *X. laevis* neural crest explants and grafts

Two-cell stage embryos were microinjected with membrane GFP or lifeact-GFP and then cultured at 15 °C until they reached an appropriate stage. The procedure followed to obtain clean neural crest was as Milet and Monsoro-Burq^70^. Neural crest explants were plated in fibronectin-covered slides to study in vitro migration. They were incubated under control and 0.5 mM BIO at time = 0. Explants were examined 8–24 h later, as indicated. For whole mount and grafts, the embryos were incubated in control and 15 mM BIO and collected when they reached the desired stage.

### Drug treatments

All drugs were prepared at the concentration indicated, in the corresponding standard media for each cell type. GSK3 inhibitor BIO (Calbiochem, 361550) was re-suspended in a stock solution of 140 mM in dimethyl sulphoxide (DMSO) and stored at −20^o^C until use for either mouse or *X. laevis* experiments. The ALK inhibitor CTB was re-suspended in a stock solution of 5.5 mM in DMSO. The ALK inhibitor AZD3463 (Selleckchem, S7106) was re-suspended in a stock solution of 20 mM in DMSO. All compounds were then further diluted in the appropriate media. For MEFs and NB cell lines, treatments were added when cells were at a confluence of at least 80%. Control treatments were performed at the corresponding DMSO concentration.

*Xenopus* embryos were incubated in 12-well plates, 20 embryos per well. For GSK3 inhibition, 15 μM BIO (Calbiochem) was added to media or as otherwise indicated. Control embryos were incubated in 0.5% DMSO in media.

### NB cell scratch assay and single-cell tracking

Cells were cultured to confluence in a 96-well ImageLock^TM^ plate (IncuCyte^TM^) in NB media. At this point, the cells were starved overnight in 2% FBS. For the scratch, a 96-pin mechanical device (WoundMaker™) was used to create homogeneous 700–800 µm wounds in the confluent monolayers after starvation. For detailed manual, see the IncuCyte® Cell Migration Kit (Essen Bioscience). The following treatments, diluted in starvation media, were applied to the cells prior to the scratch making: 1.5 µM AZD-3463, 1.5 µM CTB, 1.0 µM NVP-TAE684, 0.5 µM BIO, 1.0 µM CHIR 99021, and DMSO as control. The plates were then incubated and scanned in the IncuCyte® system at the rate of 1 scan/h for up to 36 h, but analysis was performed at the 24-h time point. Image processing and all the analysis were made using the IncuCyte® ZOOM Software. Significance was based on two-tailed *t*-test.

### Microscopy and image analysis

Live imaging was obtained using Nikon A1R. Confocal *z*-stacks were obtained using a Leica TCS SP5 DM16000. Image sequences were reconstructed using the Fiji-ImageJ analysis software.

### Data availability

The authors declare that all data supporting the findings of this study are available within the article and its supplementary information files or from the corresponding author upon reasonable request.

## Electronic supplementary material

Supplementary Information(PDF 16250 kb)

Description of Additional Supplementary Files(DOCX 16 kb)

Supplementary Movie 1

Supplementary Movie 2

Supplementary Movie 3

Supplementary Movie 4

Supplementary Movie 5

Supplementary Movie 6

Supplementary Movie 7
