# Supplementary material for: Glycogen synthase kinase 3 controls migration of the neural crest lineage in mouse and Xenopus
Source: Nat Commun. 2018 Mar 19;9:1126. doi: 10.1038/s41467-018-03512-5 (PMC5859133; doi:10.1038/s41467-018-03512-5)
Supplement: Supplementary file 1 — Supplementary Information(PDF 16250 kb) [file 41467_2018_3512_MOESM1_ESM.pdf]

SUPPLEMENTARY INFORMATION

Title: Glycogen Synthase Kinase 3 Controls Migration of the Neural Crest Lineage in Mouse and *Xenopus*

Authors: Sandra G. Gonzalez Malagon[1], Anna M. Lopez Muñoz[1,*], Daniel Doro[1,*], Triòna G. Bolger[1,*], Evon Poon[2], Elizabeth R. Tucker[2], Hadeel Adel Al-Lami[1], Matthias Krause[3], Christopher J. Phiel[4], Louis Chesler[3] and Karen J. Liu[1,6]

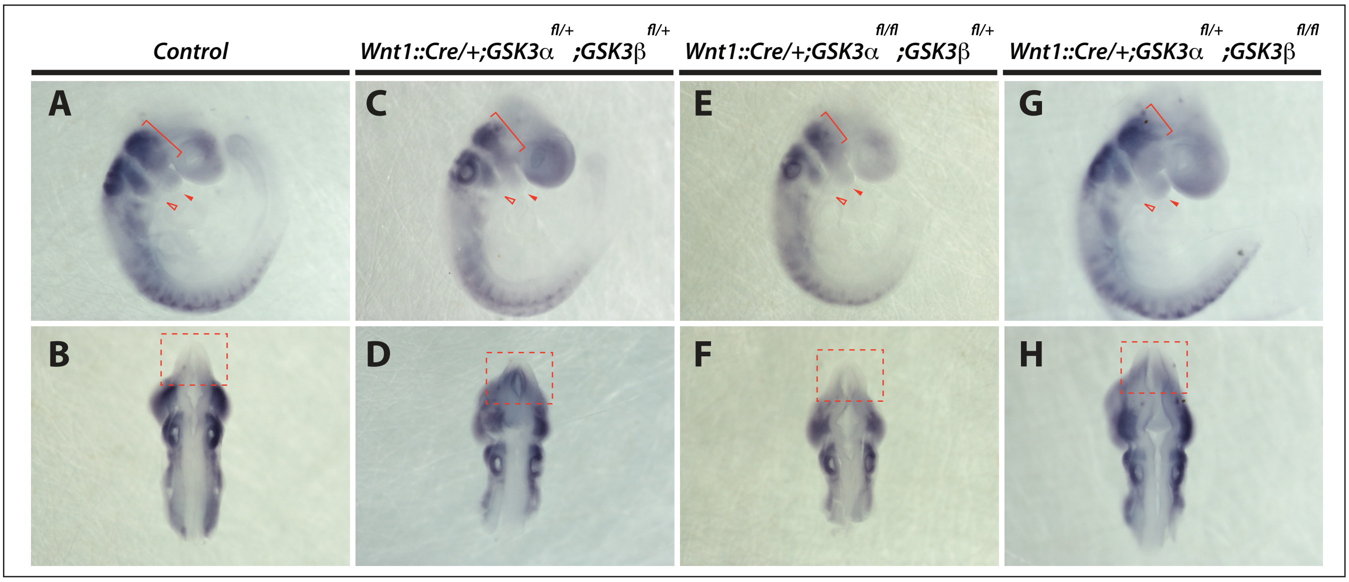

Supplementary Figure 1. Neural crest migration shows subtle pattern differences depending on specific allele deletion of GSK3. Whole mount in situ hybridization for Sox10 labels migratory neural crest in E9.5 mouse embryos. (A, C, E, G) Lateral views. (B, D, F, H) Dorsal views. (A-B) Sox10 expression in control embryos is detected in the trigeminal, branchial arches, frontonasal process and the hindbrain neural crest streams at the level of r4-r6. Note absence of Sox10 positive cells in the brain (B, red dotted square) (C, D) Neural crest specific deletion of one allele of each GSK3 isoform results in accumulation of Sox10 expressing cells in the brain (D, red dotted square) and slight reduced expression in branchial arch 1 (C, red arrowhead). (E, F) Mice carrying only one allele of GSK3β showed accumulation of Sox10 positive cells in the brain (F, red dotted square), and reduced expression in branchial arch 2 and frontonasal process (red arrowheads). (G, H) Neural crest specific deletions carrying only one allele of GSK3α, showed accumulation of Sox10 positive cells in the brain (red dotted square) and branchial arch 2 (red line arrowhead). (n≥2).

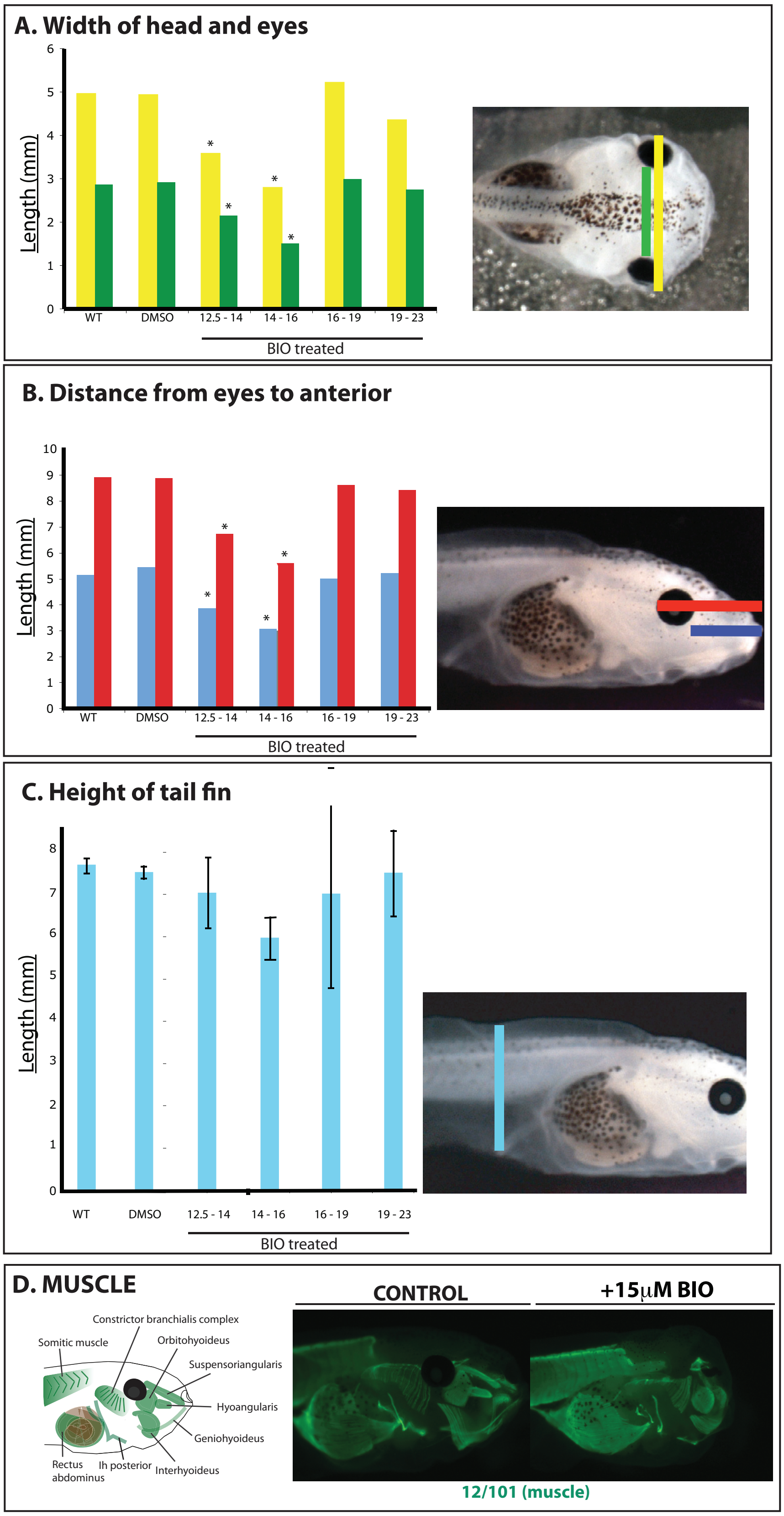

Supplementary Figure 2. Timed GSK3 inhibition during frog (Xenopus laevis) neural crest migration results in embryo phenotypic defects. (A-C) Length measurement of head structures obtained from st 45 embryos untreated and treated with GSK3 inhibitor, BIO. Note that treatments between st.12.5-14 and st.14-16, significantly affect head features such as the head width (A, yellow bars) and width between eyes (A, green bars), The same treatment timing has an effect on lateral measurements of the distance from the posterior edge of the eye (B, red bar) and the anterior edge of the eye (B, blue bar) to the anterior edge of the embryo, (C) The height of the tail fin was smaller in embryos treated from st.14 to st.16. (n=12, p>0.001) (D) Schematic of craniofacial muscle in tadpoles. 12/101 antibody staining labeling muscle revealed that BIO treatment reduced size of craniofacial muscles (green) but does not perturb patterning (n=3).

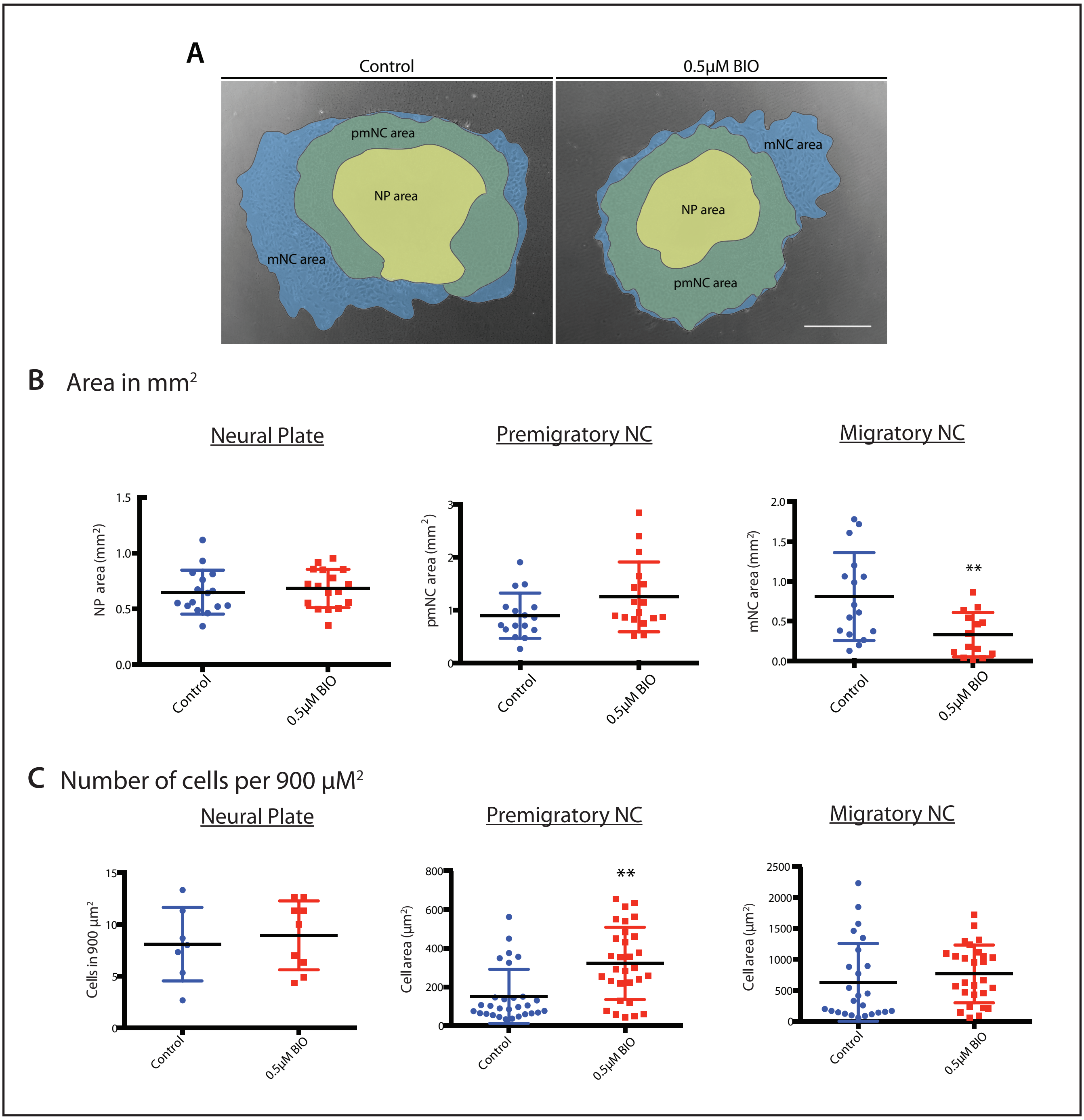

Supplementary Figure 3. GSK3 inhibition reduces migration of neural crest cells, but does not affect the number or cell size of migratory cells. (A) Neural crest explants showing in different colour shades the cell populations described. In the middle the neural plate (NP), surrounded by a cell population that appears more epithelial, the pre-migratory neural crest (pmNC), and finally in the outer ring of the explant, the migratory neural crest (mNC) population where cells appear lose and show a mesenchymal phenotype. Neural crest cells treated with BIO, show a reduced expansion in the mNC. (B) Dot plots representing the absolute values obtained from the NP, pmNC and mNC areas. (C) Dot plots showing the number of cells contained in a specific area in control and BIO treated explants. Cell area in the pmNC appeared to be increased in BIO treated explants, however in the mNC population there was no difference between BIO treated and control samples. (n>15, each dot represents an explant. **P<0.001. Scale bar=500µM.

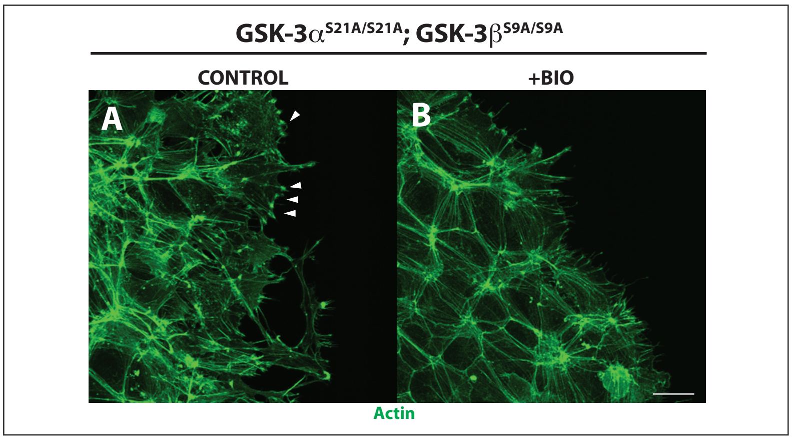

Supplementary Figure 4. Serine 9/Serine 21 phosphorylation is not necessary for normal neural crest cell morphology and knockin mice are still sensitive to BIO treatment. (A) Phalloidin staining of neural crest explants from GSK3αS21A/S21A ; GSK3βS9A/S9A embryos shows actin at the lead edge of cells and in lamellipodia (white arrowheads). (B) Treatment with BIO results in more spiky filopodial protrustions and a loss of leading edge actin. Representative images of two independent experiments. Scale bar=20μM.

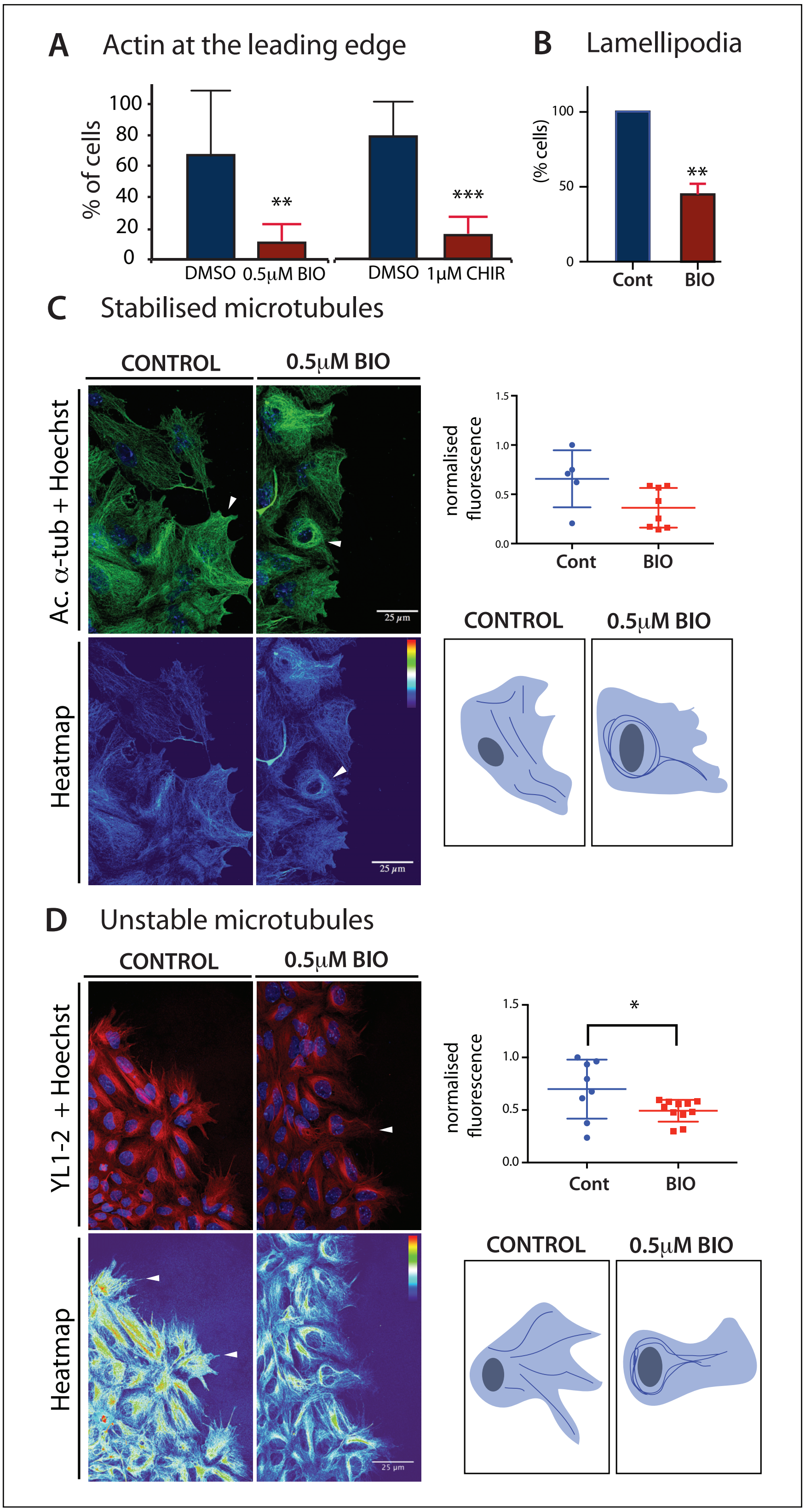

Supplementary Figure 5. Effect of GSK3 inhibition on actin, lamellipodia and microtubules. (A) Percentage of cells containing actin at the cell leading edge. GSK3 inhibition with BIO/CHIR resulted in a significant reduction of this percentage. (B) Percentage of cells that formed fan-shaped lamellipodia in control and BIO treated explants. (C) In controls (left) stabilised microtubules marked by acetylated α-tubulin are distributed throughout the cell. In BIO treated samples (right), acetylated α-tubulin staining is localized perinuclearly, with a bias towards the leading edge of the cell. Relative fluorescence is decreased in treated explants. Schematic depicts relocalisation of acetylated α-tubulin staining. (D) In controls (left) unstable microtubules, marked by YL1/2, staining is distributed throughout the cell while in BIO treated samples YL1/2 staining is perinuclear and biased toward the posterior of the cell. Relative fluorescence is significantly decreased in BIO-treated samples (*$p<0.05$). Schematic depicting relocalisation of YL1/2 staining. Representative images from two independent experiments, each dot represents an explant.

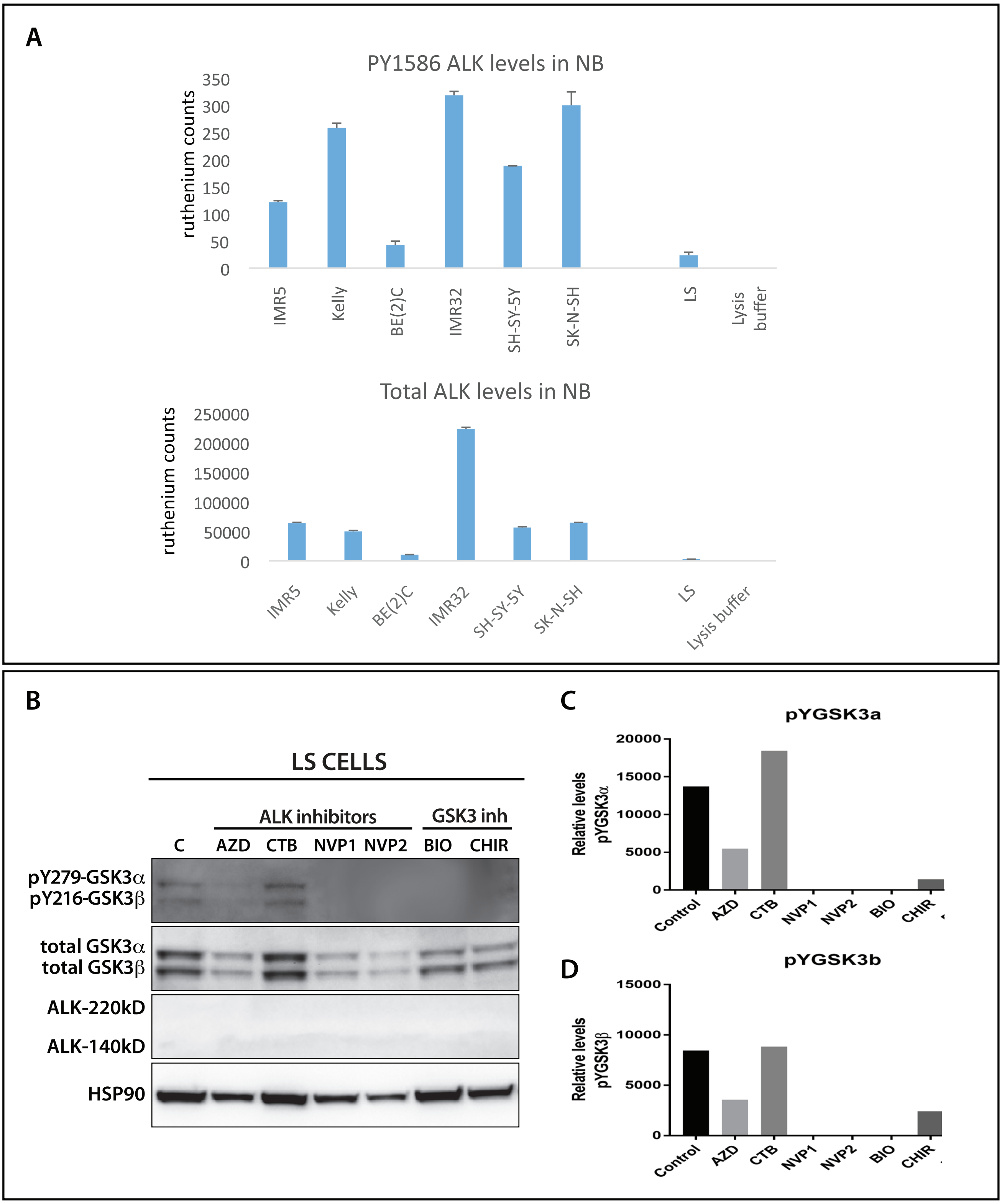

Supplementary Figure 6. pY-GSK3 profile in LS neuroblastoma cell line treated with ALK and GSK3 inhibitors. (A) Mesoscale discovery (MSD) assay showing relative levels of active ALK (pY1586) and total ALK in the neuroblastoma lines shown in Figure 7A. Note low levels of ALK in LS cells. (B) LS cells were treated with ALK inhibitors (1.5μM CTB, 1.5μM AZD-3463 and 1.0μM NVP-TAE684) and with GSK3 inhibitors (0.5μM BIO, 1.0μM CHIR99021) for 24h and analysed by western blot for pY-GSK3, total GSK3 and ALK. Analysis confirmed absence of ALK in this cell line and a lower amount of pY-GSK3 than Kelly cell line (see Figure 7D). Treatment with ALK inhibitors showed that CTB did not affect the pY-GSK3 content compared to untreated cells, however AZD and NVP seem to have reduced total GSK3 and pY-GSK3 significantly, possibly due to loss of cell viability. GSK3 inhibitors maintained total-GSK3 unaffected, however pY-GSK3 expression was not detected. (C, D) Relative levels of pY-GSK3α (C) and pY-GSK3β (D) to loading control.

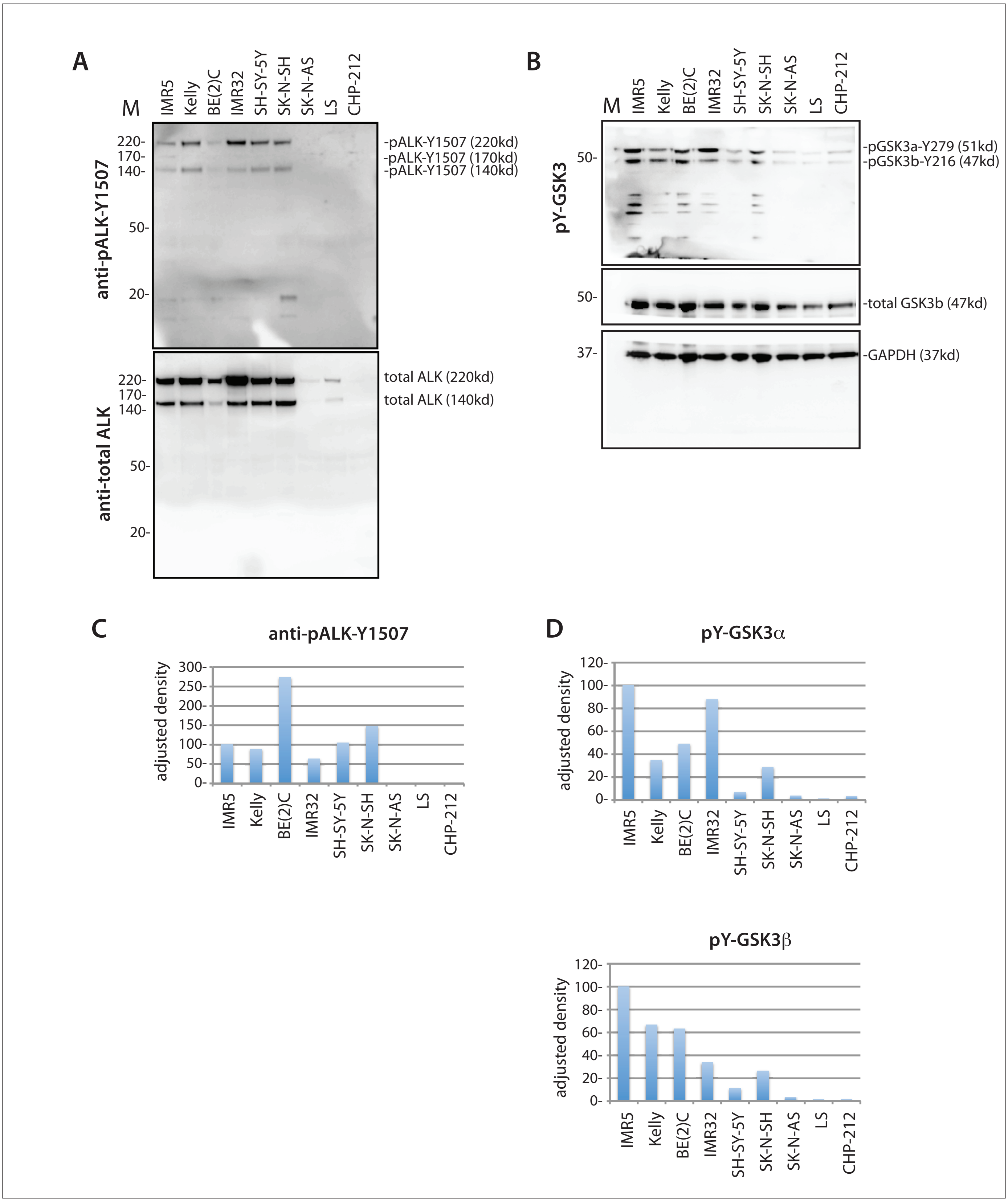

Supplementary Figure 7. Blots and quantification associated with Figure 7A
(A) Blots probed with anti-pALK-1507 (top) and anti-total ALK (bottom). (B) Blots probed with anti-pYGSK3 (top), total GSK3β (middle) and GAPDH (bottom). (C) Quantification of ALK (pALK-Y1507) normalized to GAPDH. (D) Quantification of pY-GSK3α (top) and pY-GSK3β (bottom) normalized to GAPDH.

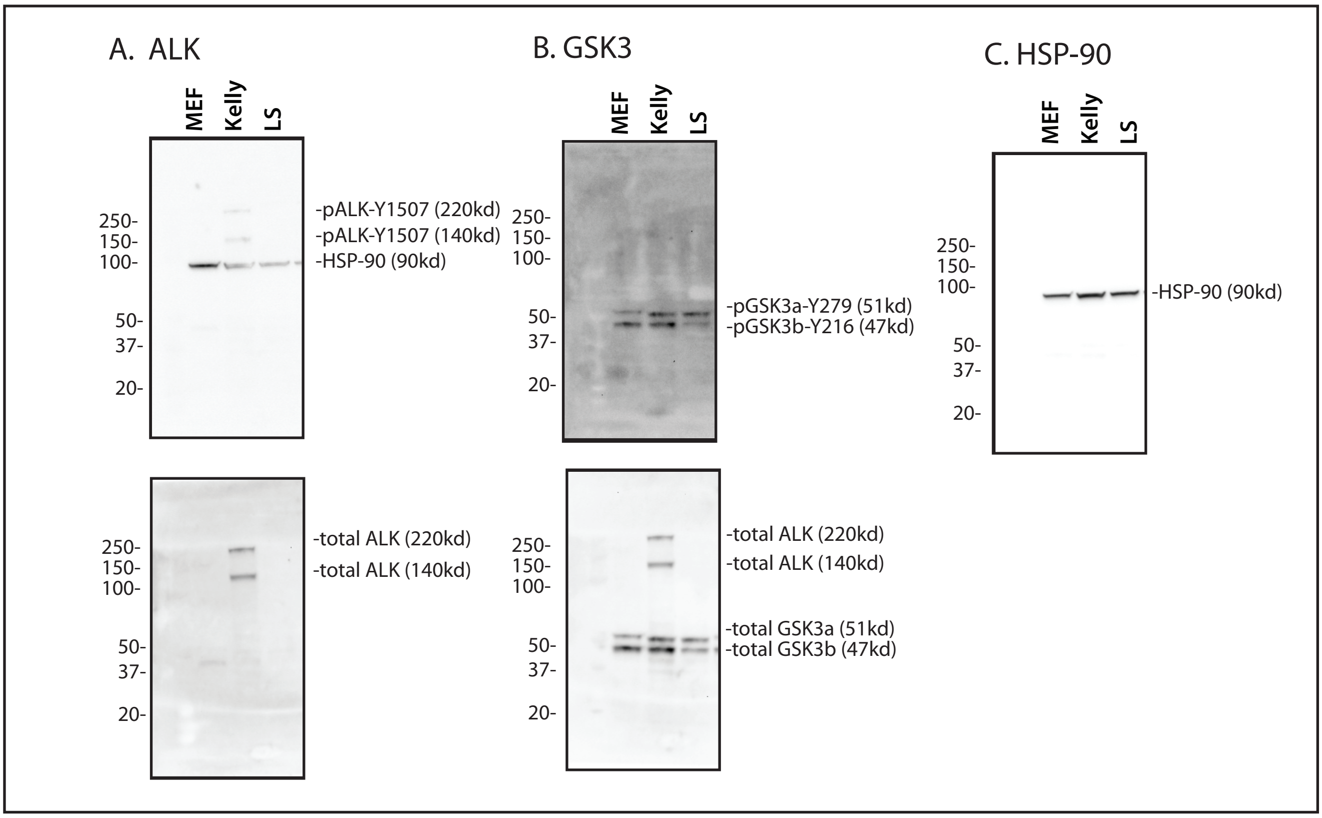

Supplementary Figure 8. Blot and quantitation associated with Figure 7B
(A) Blot probed with anti-pALK-1507 (top) and anti-total ALK (bottom). HSP-90 bands are evident as well. (B) Blot probed with anti-pYGSK3 (top), total GSK3β and total-ALK (bottom). (C) Blot probed with HSP-90.

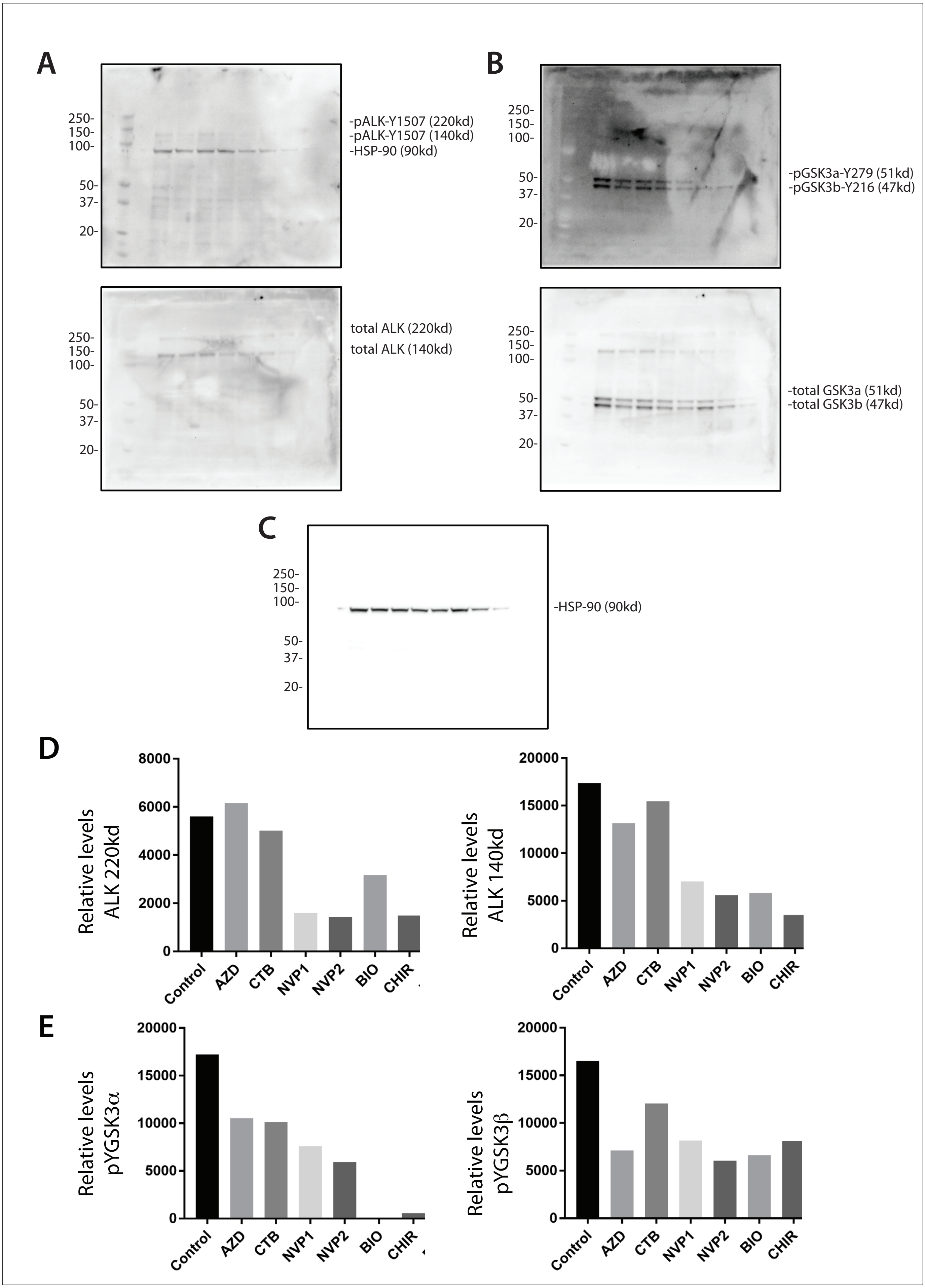

Supplementary Figure 9. Blot and quantitation associated with Figure 7C

(A) Blot probed with anti-pALK-1507 (top) and anti-total ALK (bottom). HSP-90 bands are evident as well. (B) Blot probed with anti-pYGSK3 (top), total GSK3β and total-ALK (bottom). (C) Blot probed with HSP-90. (D) Quantification of ALK-220kd (left) and ALK-140kd (right) normalized to HSP-90 levels. (E) Quantification of pY-GSK3α (left) and pY-GSK3β (right) normalized to HSP-90.

Supplementary Table 1: Mouse lines

Allele	Allele type	Reference
GSK-3α^{tm1Dral}	*GSK-3α* Knock-in of S9A mutation	McManus EJ et al., 2005[1]
GSK-3α$^{tm1a(EUCOMM)Wtsi}$	*GSK-3α* lacZ knock-in reporter and "conditional ready floxed" allele	Barrell W et al., 2012[2]
GSK-3β^{tm1Dral}	*GSK-3β* knock-in of S21A mutation	McManus EJ et al., 2005[1]
GSK-3β^{tm1Dgen}	*GSK-3β* lacZ knock-in reporter	Barrell W et al., 2012[2]
GSK-3β$^{tm1.1Ypc}$	*GSK-3β* floxed allele	He et al., [3]
pCAG::cre-ERT2	Tamoxifen inducible ubiquitous driver of cre recombinase	Hayashi & McMahon, 2002[4]
Tg(Wnt1-cre)11Rth	Wnt1 promoter driving cre recombinase	Danielian et al., 1998[5]
GT(Rosa)R26Sor$^{Tm4(ACTB-tdTomato-EGFP)Luo}$	Cre dependent membrane GFP reporter (mTomato in absence of cre)	Muzumdar et al., 2007[6]

Supplementary Table 2: Neuroblastoma cell lines

Cell line	Source, lot number	Reference
LS	DMSZ (ACC 675) lot #1	7,8
CHP-212	ATCC CRL-2273 lot #58063161	9
Kelly	DMSZ (ACC 355) lot #7	10,11
SK-N-SH	HPA lot #09/D/005	12
SH-SY-5Y	DMSZ (ACC 209) lot #12	13,14
IMR32	HPA lot #04/K/012	15,16
BE(2)C	ATCC CRL-2268 lot #10H023	17
SK-N-AS	ATCC CRL-2137 lot #58078525	18
IMR5	gift from Martin Eilers, Wurzburg	19

Supplementary Table 3. Antibodies

Immunofluorescence (IF), Immunohistochemisty (IHC) and Western Blotting (WB).

Antibody	Company/Catalogue number	Dilution for IF/IHC	Dilution for WB
mouse phospho GSK3 Tyr279/216	Millipore 05-413	1:300	1:1000
rabbit p75NTR	Millipore 07-476	1:500 IF	
rabbit total ALK D5F3	Cell Signaling Tech #3633	1:300 IF	
rabbit phospho-Y1507 ALK	Cell Signaling Tech #14678	1:200 IHC	
rabbit pFAK	Abcam 39967	1:300 IF	1:1000 WB
rabbit RAC1	Santa Cruz SC-217	1:500 IF	
mouse CDC42	Santa Cruz SC-8401	1:300 IF	
rabbit lamellipodin	provided by Krause Lab	1:200 IF	
rabbit GAPDH	Cell Signaling #2118		1:1000 WB
mouse HSP90a/b	Santa Cruz SC-13119		1:1000 WB
anti-muscle antibody	DHSB 12/101	Supernatant 1:5	
mouse IgG-Alexa 488		1:1000 IF	
mouse IgG Alexa 568		1:1000 IF	
rabbit IgG-Alexa 488		1:1000 IF	
rabbit IgG-Alexa 568		1:1000 IF	
Mouse IgG Peroxidase			1:10,000 WB
Rabbit IgG-Peroxidase			1:10,000 WB

Supplementary References

1 McManus, E. J. *et al.* Role that phosphorylation of GSK3 plays in insulin and Wnt signalling defined by knockin analysis. *The EMBO journal* **24**, 1571-1583, doi:10.1038/sj.emboj.7600633 (2005).

2 Barrell, W. B., Szabo-Rogers, H. L. & Liu, K. J. Novel reporter alleles of GSK-3alpha and GSK-3beta. *PloS one* **7**, e50422, doi:10.1371/journal.pone.0050422 (2012).

3 He, F. *et al.* Gsk3beta is required in the epithelium for palatal elevation in mice. *Dev Dyn* **239**, 3235-3246, doi:10.1002/dvdy.22466 (2010).

4 Hayashi, S. & McMahon, A. P. Efficient recombination in diverse tissues by a tamoxifen-inducible form of Cre: a tool for temporally regulated gene activation/inactivation in the mouse. *Developmental biology* **244**, 305-318, doi:10.1006/dbio.2002.0597 (2002).

5 Danielian, P. S., Muccino, D., Rowitch, D. H., Michael, S. K. & McMahon, A. P. Modification of gene activity in mouse embryos in utero by a tamoxifen-inducible form of Cre recombinase. *Curr Biol* **8**, 1323-1326 (1998).

6 Muzumdar, M. D., Tasic, B., Miyamichi, K., Li, L. & Luo, L. A global double-fluorescent Cre reporter mouse. *Genesis* **45**, 593-605, doi:10.1002/dvg.20335 (2007).

7 Corvi, R., Savelyeva, L., Amler, L., Handgretinger, R. & Schwab, M. Cytogenetic evolution of MYCN and MDM2 amplification in the neuroblastoma LS tumour and its cell line. *European journal of cancer* **31A**, 520-523 (1995).

8 Rudolph, G., Schilbach-Stuckle, K., Handgretinger, R., Kaiser, P. & Hameister, H. Cytogenetic and molecular characterization of a newly established neuroblastoma cell line LS. *Human genetics* **86**, 562-566 (1991).

9 Hay, R., Park, J.-G. & Gazdar, A. F. *Atlas of human tumor cell lines*. (Academic Press, 1994).

10 Schwab, M. *et al.* Amplified DNA with limited homology to myc cellular oncogene is shared by human neuroblastoma cell lines and a neuroblastoma tumour. *Nature* **305**, 245-248 (1983).

11 Preis, P. N. *et al.* Neuronal cell differentiation of human neuroblastoma cells by retinoic acid plus herbimycin A. *Cancer research* **48**, 6530-6534 (1988).

12 Fransson, S. *et al.* Intragenic anaplastic lymphoma kinase (ALK) rearrangements: translocations as a novel mechanism of ALK activation in neuroblastoma tumors. *Genes, chromosomes & cancer* **54**, 99-109, doi:10.1002/gcc.22223 (2015).

13 Biedler, J. L., Helson, L. & Spengler, B. A. Morphology and growth, tumorigenicity, and cytogenetics of human neuroblastoma cells in continuous culture. *Cancer research* **33**, 2643-2652 (1973).

14 Ross, R. A., Spengler, B. A. & Biedler, J. L. Coordinate morphological and biochemical interconversion of human neuroblastoma cells. *Journal of the National Cancer Institute* **71**, 741-747 (1983).

15 Mazot, P. *et al.* Internalization and down-regulation of the ALK receptor in neuroblastoma cell lines upon monoclonal antibodies treatment. *PloS one* **7**, e33581, doi:10.1371/journal.pone.0033581 (2012).

16 Tumilowicz, J. J., Nichols, W. W., Cholon, J. J. & Greene, A. E. Definition of a continuous human cell line derived from neuroblastoma. *Cancer research* **30**, 2110-2118 (1970).

17 Ciccarone, V., Spengler, B. A., Meyers, M. B., Biedler, J. L. & Ross, R. A. Phenotypic diversification in human neuroblastoma cells: expression of distinct neural crest lineages. *Cancer research* **49**, 219-225 (1989).

18 Sugimoto, T. *et al.* Determination of cell surface membrane antigens common to both human neuroblastoma and leukemia-lymphoma cell lines by a panel of 38 monoclonal antibodies. *Journal of the National Cancer Institute* **73**, 51-57 (1984).

19 Brockmann, M. *et al.* Small molecule inhibitors of aurora-a induce proteasomal degradation of N-myc in childhood neuroblastoma. *Cancer cell* **24**, 75-89, doi:10.1016/j.ccr.2013.05.005 (2013).
