# Supplementary material for: Glycogen synthase kinase 3 controls migration of the neural crest lineage in mouse and Xenopus
Source: Nat Commun. 2018 Mar 19;9:1126. doi: 10.1038/s41467-018-03512-5 (PMC5859133; doi:10.1038/s41467-018-03512-5)
Supplement: Supplementary file 2 — Description of Additional Supplementary Files(DOCX 16 kb) [file 41467_2018_3512_MOESM2_ESM.docx]

**Description of Additional Supplementary Files**

File Name: Supplementary Movie 1

Description: Movie of control neural crest explants expressing *LifeAct-GFP*. *Xenopus* embryos were injected with mRNA encoding *LifeAct-GFP* at the two-cell stage. Neural crest explants were taken at stage 17 and cultured for 8 hours before imaging. This movie corresponds to Supplementary Figure 2E-H.

File Name: Supplementary Movie 2

Description: Movie of BIO treated neural crest explants expressing *LifeAct-GFP*. *Xenopus* embryos were injected with mRNA encoding *LifeAct-GFP* at the two-cell stage. Neural crest explants were taken at stage 17 and cultured for 8 hours in 0.5µM BIO before imaging. This movie corresponds to Supplementary Figure 2I-L.

File Name: Supplementary Movie 3

Description: Movie of control mouse neural crest. Movies of migrating neural crest from controls (*GSK3a^fl/fl^; GSK3b^fl/fl^; Rosa^mTmG^*^/+^) showing normal filopodial and lamellipodial dynamics, as well as migratory behaviour.

File Name: Supplementary Movie 4

Description: Movie of GSK3 mouse mutant neural crest. Movies of mutant neural crest explants (*pCAGG::CreER^tm^; GSK3a^fl/fl^; GSK3b^fl/fl^; Rosa^mTmG^*^/+^) showing loss of motility and lamellipodial dynamics, but still showing filopodia formation.

File Name: Supplementary Movie 5

Description: Merge of control and mutant neural crest from Movie S4 and Movie S5 Experiments shown in Supplementary Movie 3 and 4 were performed in the same dish. Two sets of neural crest were plated together: *Cre* negative controls (*GSK3a^fl/fl^; GSK3b^fl/fl^; Rosa^mTmG^*^/+^) are labeled in red (membrane Tomato, mT) while *Cre* positive mutants (*pCAGG::CreER^tm^; GSK3a^fl/fl^; GSK3b^fl/fl^; Rosa^mTmG^*^/+^) are labeled in green (membrane GFP, mG).

File Name: Supplementary Movie 6

Description: Brightfield movies of control mouse neural crest cells corresponding to Figure 5I. Movies of migrating neural crest from controls showing normal filopodial and lamellipodial dynamics, as well as migratory behaviour.

File Name: Supplementary Movie 7

Description: Brightfield movies of mouse neural crest cells treated with BIO corresponding to Figure 5H. Movies of migrating neural crest from mouse explants treated with BIO showing loss of lamellipodial dynamics, as well as decreased cell movements.
